# Structural basis for protein glutamylation by the *Legionella* pseudokinase SidJ

**DOI:** 10.1038/s41467-021-26429-y

**Published:** 2021-10-26

**Authors:** Michael Adams, Rahul Sharma, Thomas Colby, Felix Weis, Ivan Matic, Sagar Bhogaraju

**Affiliations:** 1 European Molecular Biology Laboratory, 71 Avenue des Martyrs, 38042 Grenoble, France; 2 Max Planck Institute for Biology of Ageing, Joseph-Stelzmann-Straße 9b, 50931 Cologne, Germany; 3 European Molecular Biology Laboratory, Meyerhofstraße 1, 69117 Heidelberg, Germany

**Keywords:** Enzyme mechanisms, Kinases, Post-translational modifications, Cryoelectron microscopy

## Abstract

*Legionella pneumophila* (LP) avoids phagocytosis by secreting nearly 300 effector proteins into the host cytosol. SidE family of effectors (SdeA, SdeB, SdeC and SidE) employ phosphoribosyl ubiquitination to target multiple host Rab GTPases and innate immune factors. To suppress the deleterious toxicity of SidE enzymes in a timely manner, LP employs a metaeffector named SidJ. Upon activation by host Calmodulin (CaM), SidJ executes an ATP-dependent glutamylation to modify the catalytic residue Glu860 in the mono-ADP-ribosyl transferase (mART) domain of SdeA. SidJ is a unique glutamylase that adopts a kinase-like fold but contains two nucleotide-binding pockets. There is a lack of consensus about the substrate recognition and catalytic mechanism of SidJ. Here, we determined the cryo-EM structure of SidJ in complex with its substrate SdeA in two different states of catalysis. Our structures reveal that both phosphodiesterase (PDE) and mART domains of SdeA make extensive contacts with SidJ. In the pre-glutamylation state structure of the SidJ-SdeA complex, adenylylated E860 of SdeA is inserted into the non-canonical (migrated) nucleotide-binding pocket of SidJ. Structure-based mutational analysis indicates that SidJ employs its migrated pocket for the glutamylation of SdeA. Finally, using mass spectrometry, we identified several transient autoAMPylation sites close to both the catalytic pockets of SidJ. Our data provide unique insights into the substrate recognition and the mechanism of protein glutamylation by the pseudokinase SidJ.

## Abstract (web summary)

*Legionella pneumophila* (LP) employs the metaeffector SidJ to suppress the toxicity of SdeA and other LP SidE effector family members by catalysing the glutamylation of the catalytic Glu residue. Here, the authors present the cryo-EM structures of SidJ in complex with SdeA in two different states, which together with mutagenesis analysis provide insights into the substrate recognition and the mechanism of protein glutamylation by SidJ.

## Introduction

Protein ubiquitination is a fundamental posttranslational modification process that regulates a host of cellular processes including protein turnover, DNA repair, vesicular transport, innate immunity, and cell cycle^1,2^. Pathogenic bacteria do not possess any ubiquitin (Ub) system of their own, but often secrete effector proteins that hijack the host Ub pathways to evade host defense mechanisms and facilitate intracellular replication of bacteria^3^. LP secretes nearly 300 effector proteins into the host cytosol during infection, targeting a multitude of host pathways often by employing atypical biochemical activities^4,5^. The SidE family of *Legionella* effectors carry out noncanonical ubiquitination to target multiple endoplasmic reticulum (ER) membrane resident proteins, trigger ER fragmentation, and recruit the ER-derived vesicles to the *Legionella* containing vacuole (LCV)^6–8^.

SidE enzymes catalyze Ub transfer to substrate serines without the need of cellular E1 and E2 enzymes^6^. SidEs, however, need multiple steps to catalyze this noncanonical ubiquitination. In the first step, the mART domain in SidEs ADPribosylates Arg42 of Ub using NAD^+^ as a cofactor. Subsequently, the PDE domain uses ADPribosylated Ub as a substrate and performs a histidine intermediate-driven phosphoryl transfer reaction, resulting in phosphoribosyl (PR) ubiquitination of target serine residues^6–8^. A recent report found that SidEs can also transfer ubiquitin to substrate tyrosine residues through phosphoribosyl link^9^. SidEs, including the most studied member of the family SdeA, are also deemed toxic to eukaryotic cells due to the phosphoribosylation of Ub activity which renders the canonical host Ub system inactive^7^. A systematic study using yeast toxicity analysis has revealed that LP possesses at least 14 of the so-called metaeffectors which regulate the activity of some of *Legionella*’s own toxic effector proteins^10^. SidJ is one such metaeffector of LP that represses the toxicity of the SidE family and effectively rescues SidE family-induced lethality in yeast^11–13^. Interestingly, deletion of SidJ shows a more pronounced growth phenotype compared to deletion of SidEs in *Legionella* infection experiments conducted in amoeba and macrophages^11,14^. During the infection, SidEs first get secreted into the cytoplasm, where they ubiquitinate several ER-resident proteins and exert their toxicity^7,15^. Levels of SidEs in the host cytoplasm peak 30 minutes post-infection, and diminish thereafter in a SidJ-dependent manner^11,15^. SidJ deletion, therefore, results in a marked *Legionella* intracellular growth phenotype, presumably due to persistent, uncurbed toxicity of SidEs in the host cytoplasm in the absence of SidJ^12,13,16^. Although the deubiquitination (DUB) activity of SidJ remains uncorroborated^17–19^, recent studies have shown that LP contains two effectors DupA and DupB, which act as bona fide DUBs for PR ubiquitination^20,21^.

More recently, we and other groups have independently shown that SidJ catalyzes ATP-dependent glutamylation of SdeA catalytic residues Glu860 and Glu862, thereby inhibiting ADP-ribosylation of Ub, and hence PR ubiquitination^17–19,22^. The glutamylation activity of SidJ is strictly dependent upon the host protein Calmodulin (CaM), which binds to SidJ with nanomolar affinity. Structural studies of SidJ in complex with CaM have revealed that SidJ contains a kinase-like domain (KD) consisting of equivalent N and C-lobes sandwiching a canonical ATP-binding pocket. CaM binds to the C-terminal domain (CTD) of SidJ, and stabilizes the canonical pocket allosterically. Intriguingly, SidJ was also revealed to contain a noncanonical, migrated nucleotide-binding pocket within the C-lobe of the KD. Mutagenesis of residues lining both the canonical pocket and the migrated pocket resulted in the loss of SidJ’s glutamylation activity, indicating both nucleotide-binding pockets play an important role in catalysis^18,19^. It has been suggested that SidJ-mediated glutamylation occurs through a two-step mechanism: in the first step, SidJ adenylylates (attachment of AMP) SdeA, forming a transient intermediate; in the second step, free l-glutamate launches a SidJ-enabled nucleophilic attack, leading to glutamylation and causing AMP release^19^. But the basis for the specificity of SidJ towards SidE enzymes, and the precise roles of the two nucleotide-binding pockets in coordinating the catalysis remain unknown. Black et al. have hypothesized that the canonical pocket of SidJ is responsible for carrying out initial ATP hydrolysis, coupled to the adenylylation of Glu860 of SdeA, whereas the migrated pocket is suggested to be responsible for the glutamylation reaction^19^. However, Sulpizio et al. have found that mutating residues in the migrated pocket also disrupted the adenylylation of Glu860 of SdeA, and argued that the migrated pocket could be an allosteric nucleotide-binding site necessary to stabilize the canonical pocket, which executes both adenylation and glutamylation reactions^18^.

Here, we trapped the pre-glutamylation reaction intermediate of the SidJ/CaM-SdeA complex for structural studies by introducing a single point mutation in SidJ. We purified a stable complex of adenylylated SdeA bound to SidJ, and using cryogenic electron microscopy (cryo-EM), we determined a 2.9 Å structure of this complex. The structure reveals that the adenylylated E860 residue of SdeA is inserted into the migrated pocket of SidJ, and is poised for glutamylation. We have also determined the post-catalytic state structure of SidJ/CaM and SdeA, in which we captured apo-SidJ bound to glutamylated SdeA, likely poised for the next adenylylation and glutamyl chain extension reactions. Both PDE and mART domains of SdeA interact with SidJ, explaining why a truncation in the PDE rendered SdeA resistant to SidJ in previous yeast toxicity rescue experiments^12^. Our structure-based mutational analysis revealed that SidJ, despite adopting a pseudokinase fold, uses its noncanonical migrated pocket for glutamylation of its substrates. Furthermore, using mass spectrometry, we found that SidJ undergoes autoAMPylation on specific lysine and glutamate residues proximal to both catalytic pockets.

## Results

### Trapping SidJ during the glutamylation of SdeA

**Fig. 1:**
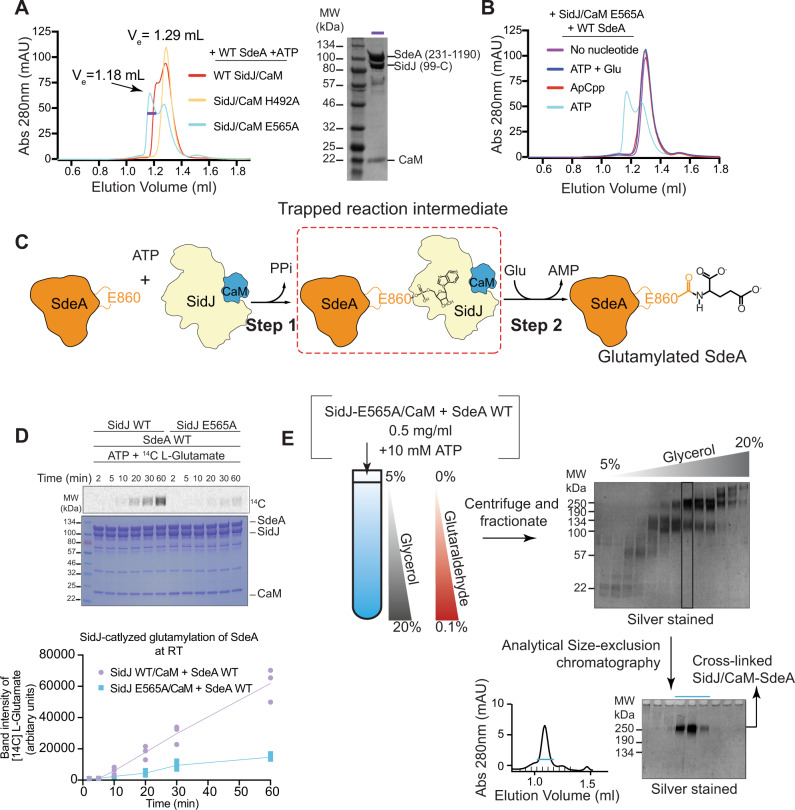
SidJ E565A/CaM and SdeA form a stable reaction intermediate complex. **A** Size-exclusion chromatography (SEC) profiles of SidJ/CaM and SdeA in the presence of ATP. The highlighted fraction in the SidJ/CaM E565A + SdeA sample is shown on SDS-PAGE (right). **B** SEC profiles of SidJ/CaM E565A + SdeA in the presence of various cofactors. **C** Schematic representation of the reaction scheme of SidJ-mediate glutamylation of SdeA highlighting the trapped adenylylated reaction intermediate between SidJ/CaM and SdeA. **D** Time course experiment measuring the incorporation of [^14^C]-Glu into SdeA catalyzed by SidJ using SidJ WT, SidJ E565A. Samples were separated by SDS-PAGE and visualized by Coomassie stain and autoradiography (Top). The experiment was done in triplicates and [^14^C]-bands were quantified and plotted (Bottom). Purple corresponds to WT, blue corresponds to the E565A mutant. Individual measurements are shown. **E** Flow chart of the sample preparation process of SidJ E565A/CaM + SdeA for Cryo-EM. From left to right, samples are incubated and then cross-linked via GraFix method. Fractions are analyzed via SDS-PAGE and further purified via SEC.

SidJ and SdeA did not show any detectable co-elution when assayed using in cellulo co-IPs, indicating a transient nature of the interaction possibly limited to the moment of catalysis (Supplementary Fig. 1A). We hypothesized that a mutant form of SidJ might be able to alter the glutamylation reaction kinetics to make the interaction between SidJ and SdeA less transient. To test this, we mutated a number of SidJ residues both in the canonical pocket (D542A, K367A, Q350A, T353A, K370A, and Y452A) (Supplementary Fig. 1B) and in the migrated pocket (E565A and H492A) (Supplementary Fig. 1C), and purified these SidJ mutants in complex with CaM. We then used these individual mutant SidJ-CaM complexes in an in vitro reaction with SdeA (residues 231 to 1190 containing both PDE and mART domains) and ATP and subjected the reaction mixture to analytical size-exclusion chromatography (Supplementary Fig. 1D). As expected, the WT SidJ-CaM complex and SdeA did not form a heterotrimeric complex under these conditions and eluted as heterodimer and monomer respectively (Supplementary Fig. 1D, E). Among the SidJ mutants tested, a mutant of the migrated pocket, SidJ E565A-CaM, incubated with SdeA and ATP eluted in two separate but overlapping peaks (Fig. 1A). The elution peak corresponding to the estimated molecular weight of ~200 kDa contained stoichiometric amounts of SdeA, SidJ, and CaM (Fig. 1A) indicating the formation of a ternary complex. Notably, SidJ E565A-CaM and SdeA did not form the ternary complex under similar conditions when l-glutamate was added to the reaction mix or when we replaced ATP in the reaction mix with the non-cleavable ATP analog ApCpp (Fig. 1B).

In WT SidJ, hydrolysis of ATP leads to adenylylation of the target glutamate of SdeA, resulting in the formation of an acyl-AMP moiety (Fig. 1C). Subsequently, the amino group of a free l-glutamic acid molecule launches a SidJ-enabled nucleophilic attack on the acyl-AMP intermediate, resulting in glutamylation of the target residue via an isopeptide bond. Our data (Fig. 1A, B) indicates that the SidJ E565A-CaM-SdeA heterotrimeric complex is likely a reaction intermediate that occurs after ATP hydrolysis but before glutamylation by SidJ. Surprisingly, SidJ E565A glutamylates SdeA to the same extent as WT in our in vitro endpoint glutamylation assays (Supplementary Fig. 1F) performed at 37 °C. Our time course glutamylation experiment performed at room temperature revealed that SidJ E565A is considerably slower in glutamylating SdeA compared to SidJ WT (Fig. 1D). To study this pre-glutamylation complex using cryo-EM, we applied gradient fixation (GraFix)^23^ on the SidJ E565A-CaM-SdeA complex and purified the cross-linked complex further using analytical size-exclusion chromatography (Fig. 1E). The purified SidJ E565A-CaM-SdeA complex elutes in a single size-exclusion peak and runs as one cross-linked species in SDS-PAGE (Fig. 1E). Importantly, the cross-linked SidJ E565A-CaM-SdeA complex is still active as assayed through in vitro glutamylation reactions (Supplementary Fig. 1F) indicating that GraFix does not completely incapacitate the complex.

### Overall structure of SidJ-CaM-SdeA ternary complex in pre-glutamylation state

**Fig. 2:**
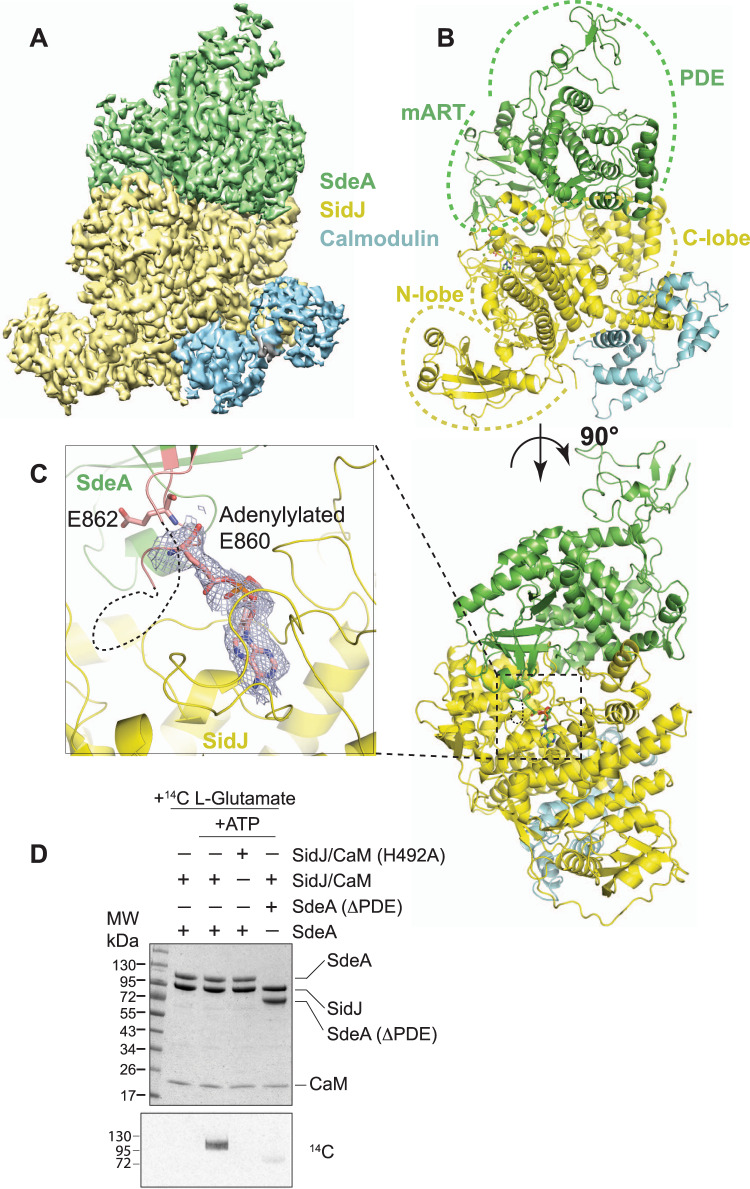
Adenylylated SdeA E860 binds to the migrated pocket of SidJ. **A** Cryo-EM map of SidJ E565A (Yellow), Calmodulin (Teal), and SdeA (Green). **B** Overall structure of the reaction intermediate SidJ E565A/CaM/SdeA heterotrimer. Both N- and C-lobe of SidJ (Yellow), as well as mART and PDE lobes of SdeA (Green) are highlighted, with Calmodulin (Teal) bound to the C-terminus of SidJ. **C** Closer view of the migrated nucleotide-binding pocket of SidJ (Yellow), and adenylylated SdeA E860 (Stick representation, Green) inserted. The cryo-EM density of E860 of SdeA and AMP is shown in the mesh. SdeA catalytic loop is colored in salmon. E862, another glutamylation target of SidJ is also shown. **D** Incorporation of [14 C]-Glu into SdeA with and without its PDE domain present. Reaction components were separated by SDS-PAGE and either visualized by Coomassie stain (Top) or autoradiography (Bottom).

The purified SidJ E565A/CaM-SdeA complex was analyzed by single-particle cryo-EM, yielding a density map with a nominal resolution of 2.9 Å (Supplementary Fig. 2 and Supplementary Table 1). The obtained map was used to rigid body fit both SidJ/CaM (PDB:6oqq)^17–19,22^ and SdeA 231-1190 (PDB:5yij)^24–27^ and refined. The C-terminal region of SdeA corresponding to residues 905 to 1190 is not resolved in the EM map (Supplementary Fig. 2) and the alpha-helical lobe (AHL corresponding to residues 591–757) of the mART domain is also not adequately resolved in the map. The refined structure contains one molecule each of SdeA, SidJ, and CaM with the adenylylated E860 of SdeA pointing directly into the migrated pocket of SidJ (Fig. 2A–C). The isolated SidJ/CaM structure superimposes well with the SidJ/CaM in complex with SdeA with a mean r.m.s.d of 0.5 Å over 744 C-α atoms (Supplementary Fig. 3A). The SdeA structure that was previously resolved through X-ray crystallography also superimposes well with SdeA in complex with SidJ with a mean r.m.s.d of 1.3 Å over 665 C-α atoms (Supplementary Fig. 3B). The overall structure of the SidJ/CaM-SdeA heterotrimer resembles a triangular pyramid with the apex formed of the SdeA PDE domain and the base of the pyramid formed by the “back-face” of SidJ/CaM (Fig. 2A, B). The interface of SidJ and SdeA contains both hydrophobic and polar interactions with a combined buried surface area of ~2100 Å^2^. CaM in the SidJ/CaM-SdeA complex adopts a similar conformation as seen in the SidJ/CaM complex alone and is not involved in any direct contact with SdeA. The interaction between SidJ and SdeA is mediated through multiple domains of both SidJ and SdeA. The PDE domain and mART domain of SdeA interact with the C-lobe of SidJ KD (Fig. 2B). Intriguingly, the N-lobe of SidJ KD and the canonical pocket of SidJ, which is implicated in the catalysis, do not make any contact with SdeA (Fig. 2B). Accordingly, the canonical pocket of SidJ also does not contain any bound nucleotide. Glutamylation target residue E860 of SdeA is clearly seen adenylylated and inserted into the migrated pocket of SidJ. This indicates that the trapped SidJ/CaM-SdeA complex represents a state of catalysis after the adenylylation of SdeA and before the glutamylation. Interestingly, there is no discernible density for adjacent residues 854–859 of the mART catalytic loop of SdeA (Fig. 2C). This implies that the specificity of SidJ towards E860 of SdeA is achieved through other sites of SidJ–SdeA interactions distant from the catalytic site and likely independent of the sequence immediately surrounding the target glutamate. Accordingly, a SdeA ΔPDE construct (spanning residues 531–1190) containing the intact mART domain could only be glutamylated negligibly by SidJ, indicating that SidJ relies on interactions with both PDE and mART domains of SdeA to specifically glutamylate E860 of SdeA (Fig. 2D). Residue H492 in SidJ which coordinates the α-phosphate group of AMP in the migrated pocket (Supplementary Fig. 1C) was shown in previous studies to be essential for SidJ catalysis^18,19^, hence we used SidJ H492A as a negative control in our biochemical assays. Consistent with the involvement of the SdeA PDE domain in interaction with SidJ, previous yeast toxicity experiments have also observed that SidJ could not rescue the toxicity of the SdeA construct lacking part of the PDE domain^12^.

### Characterization of SidJ–SdeA interface

**Fig. 3:**
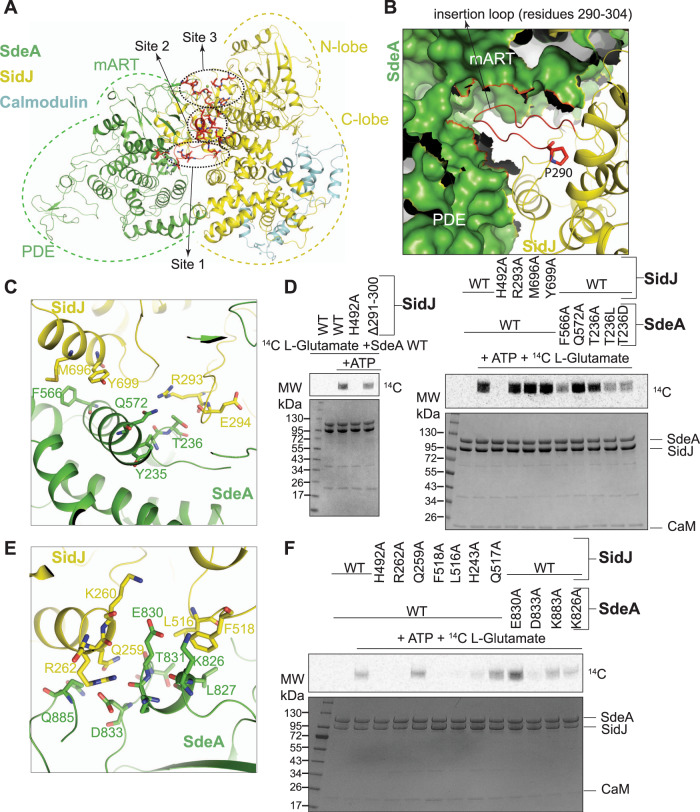
SidJ and SdeA form an extensive binding interface. **A** Overview of SidJ/CaM and SdeA intermediate complex showing the three binding sites between SidJ and SdeA. Site 1 being the loop insertion site (Shown in 3B, C), site 2 being the mART site (Shown in 3E), and site 3 being the migrated pocket site (Shown in 4A). **B** The insertion loop of site 1 (Red) of SidJ inserting itself into the cleft between SdeA’s PDE and mART lobes (Shown in green, surface representation). SidJ Proline 290 is shown in stick representation. **C** Detailed view of the SidJ–SdeA interaction site 1—the insertion loop of SidJ (Yellow) and SdeA (green) with key residues shown. **D** Incorporation of [^14^C]-Glu into SdeA catalyzed by SidJ, with and without the insertion loop (Left) and using mutations on both SidJ and SdeA from site 1 (Right). Samples were separated by SDS-PAGE and visualized by Coomassie stain (Bottom) or autoradiography (Top). **E** Detailed view of the SidJ–SdeA interaction site 2—the interactions between SidJ C-lobe (Yellow) and SdeA mART, with key residues shown. **F** Incorporation of [^14^C]-Glu into SdeA catalyzed by SidJ using mutations on both SidJ and SdeA from site 2. Samples were separated by SDS-PAGE and visualized by Coomassie stain (Bottom) or autoradiography (Top).

The interface of SidJ and SdeA can be divided into three major contact sites, spanning multiple domains of both proteins (Fig. 3A). Site 1 involves an insertion loop in SidJ spanning residues 290 to 304, which protrudes outwards from the surface of SidJ and inserts into the groove between the PDE and the mART domains of SdeA (Fig. 3B). Interestingly, a previous yeast screening experiment has found that mutation of P290, which is involved in the formation of a sharp kink at the start of this insertion loop, renders SidJ deficient in rescuing the toxicity of SdeA^13^ (Fig. 3B). SidJ R293 at the tip of this insertion loop participates in a hydrogen-bonding network involving SdeA Q572 and the backbone carbonyl of SdeA Y235 in its PDE domain (Fig. 3C). Mutating R293 of SidJ did not affect the glutamylation of SdeA as assayed through in vitro glutamylation assays using C^14^ labeled l-glutamate (Fig. 3D, right). Deletion of the extending insertion loop in SidJ (SidJ Δ291–300) resulted in only a minimal reduction of glutamylation in vitro, indicating that this loop does not play a critical role in SidJ–SdeA interaction and other interaction sites may complement in its absence (Fig. 3D, left). Site 1 of SidJ–SdeA interaction also involves F566 of SdeA making hydrophobic contacts with M696 and Y699 of SidJ (Fig. 3C). Additionally, T236 of SdeA is within hydrogen-bonding distance to the backbone carbonyl of E294 of SidJ (Fig. 3C). Mutating T236 or F566 of SdeA resulted in a strong reduction of SidJ-mediated glutamylation in vitro (Fig. 3D, right).

Site 2 of interaction between SidJ and SdeA mainly involves a short helical insertion in the mART domain of SdeA spanning residues 825–833, mediating interactions with the C-lobe of SidJ KD (Fig. 3A–E). Specifically, these interactions include two salt bridges between R262, K260 of SidJ and D833, E830 of SdeA, respectively. Site 2 also includes a tight hydrogen-bonding network with the side chains of residues SidJ Q259, SdeA Q885, and the backbone carbonyl of SdeA T831. In addition, residues SidJ F518, L516 pack against SdeA L827 and the aliphatic part of SdeA K826 constituting a hydrophobic interface between SidJ and SdeA. Single point mutations of a few residues involved in Site-2 resulted in a loss of glutamylation activity in vitro (Fig. 3F).

**Fig. 4:**
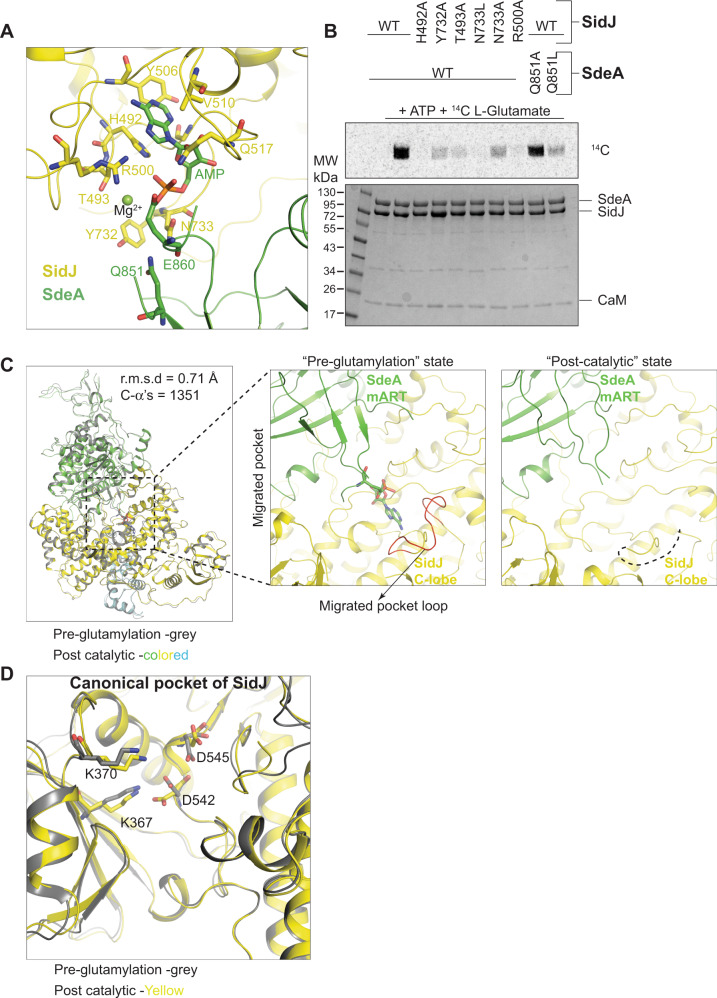
SidJ catalyzes glutamylation in the migrated pocket. **A** Detailed view of the SidJ–SdeA Interaction site 3- adenylylated E860 of SdeA (Green) inserted into the migrated pocket of SidJ (Yellow), with key residues shown. **B** Incorporation of [^14^C]-Glu into SdeA catalyzed by SidJ using mutations on both SidJ and SdeA from site 3. Samples were separated by SDS-PAGE and visualized by Coomassie stain (Bottom) or autoradiography (Top). **C** Structural comparison between SidJ E565A (Yellow)/CaM + SdeA (Green) in its pre-glutamylation and post-catalytic states. Highlighted is a loop of SidJ (Red) located in the migrated pocket that undergoes structural change post-catalysis. **D** Overlay of SidJ canonical pocket in pre-glutamylation (Gray) and post-catalytic (Yellow) state, with key residues highlighted.

The migrated pocket of SidJ is bound tightly to the adenylylated E860 of SdeA, and forms site 3 of interaction between SidJ and SdeA (Fig. 4A). AMP assumes a similar conformation in SidJ’s migrated pocket as it does in the previously described crystal structures of SidJ/CaM alone^19^ (Supplementary Fig. 4A). Surprisingly, there are only a few contacts between SidJ and SdeA in site 3 apart from the ones involving the AMP moiety covalently linked to SdeA. A key hydrogen-bonding network involving Q851 of SdeA, the backbone carbonyl of E860 of SdeA, and Y732, N733 of SidJ positions the glutamylation target residue SdeA E860 in the migrated pocket of SidJ (Fig. 4A). Mutating residues involved in this hydrogen-bonding network compromises SdeA glutamylation by SidJ (Fig. 4B). A Mg^2+^ ion and the side chain of SidJ R500 engage in tight coordination with the α-phosphate of the AMP, likely increasing the reactivity of the acyl-AMP intermediate towards the amino group of the incoming glutamate. Surprisingly, the introduced SidJ mutation E565A in the SidJ/CaM-SdeA complex did not cause any noticeable changes in the migrated pocket of SidJ compared to previously resolved SidJ structures (Supplementary Fig. 4B). Although we are not able to pinpoint why SidJ E565A binds SdeA less transiently in this reaction intermediate compared to SidJ WT, we speculate that the altered microenvironment of SidJ’s migrated pocket in E565A increases the residence time of adenylylated SdeA on SidJ (Supplementary Fig. 4C). This speculation is corroborated by the kinetics experiment (Fig. 1D) showing SidJ E565A to possess substantially lower substrate glutamylation activity compared to WT SidJ, especially in lower temperatures at which we prepared our protein complexes.

### Structural evidence for SdeA glutamylation at the migrated pocket of SidJ

The SidJ/CaM-SdeA pre-glutamylation state structure described above strongly supports the hypothesis that SidJ’s migrated pocket is the site of SdeA E860 glutamylation. We further probed the catalytic role of the migrated pocket by exploiting the GraFix cross-linked SidJ/CaM-SdeA complex sample which still performs glutamylation reaction (Supplementary Fig. 1F). We added l-glutamate to the prepared SidJ/CaM-SdeA pre-glutamylation complex right before applying the sample to the EM grid and analyzing it by single-particle cryo-EM (Supplementary Fig. 5 and Supplementary Table 1). Interestingly, after 3D classification of the particles, we observed two well-resolved particle classes, Class I (15% of total particles) contained both SidJ/CaM and SdeA well resolved and Class II (17% of total particles) represented only SidJ/CaM with discernible density while SdeA was found to be disordered, presumably dissociated from SidJ but still tethered to it due to GraFix (Supplementary Fig. 5). We pursued the processing of Class I particles to obtain a 3.7 Å reconstruction of SidJ/CaM-SdeA in a post-catalytic (PC) state (Supplementary Fig. 5). Overall, the PC state structure of SidJ/CaM-SdeA superposes well with the pre-glutamylation state structure with an r.m.s.d of 0.71 Å over 1351 C-α atoms with noticeable differences only in the migrated pocket region of SidJ and the mART domain of SdeA (Fig. 4C). Interestingly, post-glutamylation, the SidJ loop (residues 492–501) making up the migrated pocket becomes disordered in the structure, while the canonical pocket architecture remains intact (Fig. 4C, D). This observation argues against the role of migrated pocket as an allosteric center that stabilizes the canonical pocket by binding to AMP. Consistent with the idea that AMP releases upon SdeA glutamylation^19^, the electron density corresponding to AMP in SidJ’s migrated pocket is missing in the post-catalytic state structure of SidJ/CaM-SdeA (Fig. 4C). There is, however, no electron density in the post-catalytic SidJ/CaM-SdeA structure for the added l-glutamate near SdeA E860, likely because of the flexible nature of the modification.

### Characterization of autoAMPylation of SidJ

**Fig. 5:**
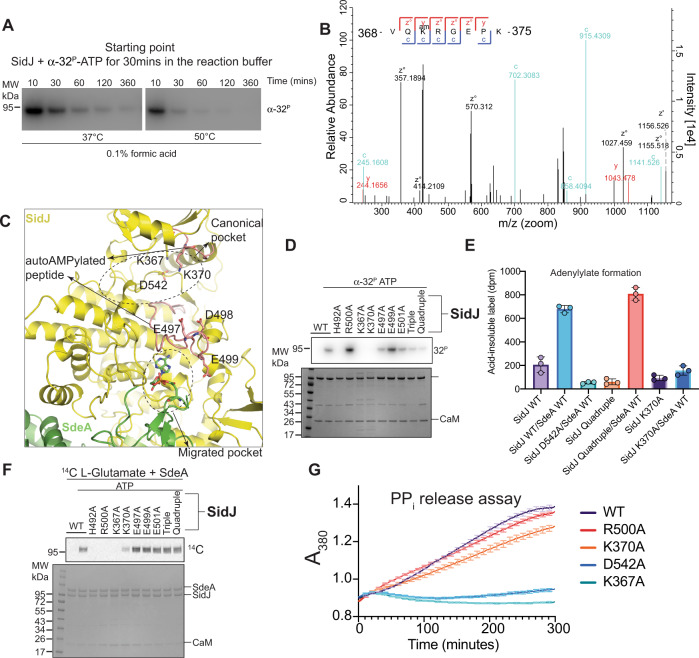
Characterization of SidJ autoAMPylation. **A** Time course of SidJ autoAMPylation in an acidic environment at different temperatures. Reactions were performed using SidJ WT + CaM and α-[32 P]-ATP. Samples were separated by SDS-PAGE and visualized by autoradiography. **B** ETD fragmentation spectrum of SidJ peptide VQKRGEPK, with the AMPylation site K370 highlighted. AutoAMPylation reactions of SidJ WT were analyzed using LC–MS/MS. **C** Detailed view of SidJ autoAMPylation sites, with peptides identified in mass spectrometry shown in red, and modification sites highlighted. **D** AutoAMPylation of SidJs. CaM + SidJ WT or indicated mutants were reacted with α-[32 P]-ATP. Samples were separated by SDS-PAGE and visualized by Coomassie stain (Bottom) and autoradiography (Top). **E** Measuring α-[^32^P]-AMP incorporation into various SidJ and SdeA mutants. The samples indicated were reacted with α-[^32^P]-ATP and TCA precipitated followed by scintillation counting to measure the AMPylation levels. Each condition was measured in triplicates. Error bars denote standard deviation. **F** Incorporation of [14 C]-Glu into SdeA catalyzed by SidJ using SidJ WT and mutants of the autoAMPylation sites. Samples were separated by SDS-PAGE and visualized by Coomassie stain (Bottom) and autoradiography (Top). **G** Pyrophosphate release assay with SidJ and mutants indicated. The assay was performed using the EnzCheck™ pyrophosphate assay kit (Thermo Fischer Scientific). The kit components (see methods) react with the PPi in solution and release ribose 1-phosphate and 2-amino-6-mercapto-7-methylpurine which shows an absorption peak at 380 nm. The average of triplicate measurements is plotted, with standard deviation bars being shown in 10-min intervals.

Having gained structural insights into the second step of SidJ-mediated SdeA glutamylation, we sought to understand how the first step (SdeA adenylylation) of SidJ catalysis occurs. Previous studies have proposed that SdeA adenylylation is catalyzed by the canonical pocket of SidJ^18,19^. Interestingly, Sulpizio et al. have reported that SidJ possesses autoAMPylation activity^17^. It was also shown that mutating both canonical pocket (D542) and migrated pocket (H492) residues renders SidJ deficient in this autoAMPylation activity^18^. Since SidJ autoAMPylation is similar in nature to SdeA adenylylation, which also involves the addition of AMP moiety, we probed the nature of autoAMPylation of SidJ and its relevance in SidJ catalysis. We first checked if SidJ autoAMPylation occurs in *cis* or *trans*; we used differently tagged SidJ proteins and found that SidJ autoAMPylates only in *cis* (Supplementary Fig. 6A). Next, we tested the stability of the autoAMPylated SidJ and found that it is both heat and acid-labile (Fig. 5A). This indicates that the AMP is attached to SidJ through an unstable bond and autoAMPylated SidJ is transient in nature. Interestingly, compared to SidJ WT, we noticed that a migrated pocket mutant SidJ R500A exhibits a marked increase in SidJ autoAMPylation and also acyl adenylylate formation with SdeA (Supplementary Fig. 6B). Using both SidJ WT and R500A proteins, we then aimed to identify the autoAMPylation sites in SidJ using mass spectrometry. We reacted SidJ (WT or R500A) with ATP and subjected the proteins to tryptic digestion followed by LC–MS/MS analysis. Surprisingly, we detected several transient autoAMPylation sites on residues close to the two catalytic pockets of SidJ in both SidJ WT and SidJ R500A proteins (Fig. 5B, C and Supplementary Fig. 7). In agreement with the radioactive assays (Supplementary Fig. 6B and Fig. 5A), we obtained more spectra for AMPylated peptides in SidJ R500A compared to WT SidJ (Fig. 5C, D and Supplementary Fig. 7). Yet, we found two peptides of SidJ that are AMPylated in both WT and the R500A mutant (Fig. 5B, C and Supplementary Fig. 7). One of these peptides (368-VQKRGEPK-375) lines the canonical pocket of SidJ (Fig 5B–D) and we could precisely localize the site of AMPylation on this peptide to the residue K370 using Electron Transfer Dissociation (ETD) fragmentation (Fig. 5B and Supplementary Fig. 7A). AutoAMPylation of SidJ was also detected in a peptide (480- EGIMFPQLADIFHTHFGEDEREDK-503 (Supplementary Fig. 7B, C and Supplementary Fig. 8A) forming a long loop (referred to as bridging peptide from now on) that originates at the canonical pocket and extends into the migrated pocket (Fig. 5C). We obtained several higher-energy collisional dissociation (HCD) fragmentation spectra of the AMPylated bridging peptide in SidJ R500A mutant (Supplementary Fig. 7B, C). Although we could not precisely localize the modification to a specific residue in the bridging peptide (because HCD fragmentation breaks off AMP from the modified residue, see Methods), we could narrow down the site through ETD fragmentation to a three residue stretch 497-EDE-499 close to the migrated pocket of SidJ (Supplementary Fig. 8A). Interestingly, SidJ autoAMPylation on these acidic residues in the bridging peptide is chemically identical to SidJ-mediated SdeA adenylylation on E860. In reactions containing both SidJ and SdeA, we could also obtain an HCD spectrum showing the AMPylation of the catalytic peptide in SdeA with likely target residue as E862 (Supplementary Fig. 8B), providing the strongest evidence yet that SdeA is adenylylated in the course of glutamylation.

We then performed site-directed mutagenesis on these SidJ autoAMPylation sites to probe if they affect autoAMPylation levels of SidJ and glutamylation of SdeA. Since there is a cluster of glutamates (E497, E499, E501, and E565) concentrated in and around the bridging peptide of SidJ (Supplementary Fig. 9), we decided to mutagenize all these glutamates individually and combined. Mutating single glutamates in the bridging peptide did not result in the decrease of autoAMPylation, but mutating the cluster of glutamates resulted in a marked reduction in SidJ autoAMPylation (Fig. 5D). Interestingly, mutating the canonical pocket autoAMPylation residue K370 in SidJ completely abolishes autoAMPylation, indicating that the AMPylation of K370 precedes that of the bridging peptide residues (Fig. 5D). Surprisingly, none of the bridging peptide mutations in SidJ affected the adenylylation of SdeA but mutating SidJ K370 resulted in a dramatic reduction of SdeA adenylylation activity (Fig. 5E). Accordingly, SidJ K370A displayed a marked decrease in SdeA glutamylation activity compared to SidJ WT, while the bridging peptide mutants did not (Fig. 5F). We next checked if the effect of SidJ K370A mutation on autoAMPylation and glutamylation activities is due to a possible defect in ATP hydrolysis. The ATP hydrolysis activity of SidJ K370A is comparable to that of WT as revealed in our in vitro pyrophosphate release assays (Fig. 5G). Mutating catalytic Lys367 and Glu542 in the canonical pocket of SidJ completely abolishes the ATP hydrolysis activity (Fig. 5F), indicating a clear role of canonical pocket in ATP hydrolysis by SidJ. Based on our data, autoAMPylation of glutamates in the bridging peptide of SidJ seem to be by-products of the reaction catalyzed by SidJ, although other roles such as self-regulation cannot be ruled out. Importantly, we show that the autoAMPylation target residue SidJ K370 is important for both SdeA adenylylation and glutamylation. Although our data implies that the autoAMPylation of SidJ K370 could be one of the precursors of SdeA adenylylation and glutamylation, other roles for SidJ K370 in catalysis cannot be excluded. Further experimentation into autoAMPylation of SidJ is necessary in order to understand its possible relevance in catalysis or autoregulation.

## Discussion

Here, we determined the cryo-EM structure of SidJ bound to SdeA trapped at an intermediate step of catalysis. The C-lobe of the kinase-like domain of SidJ makes extensive contacts with both the mART and the PDE domains of SdeA, with three major sites of contact between the two proteins. The residues of SdeA important for binding to SidJ are also mostly conserved or have permissible mutations in other SidE homologs (Supplementary Fig. 10). All three major sites of SidJ–SdeA interaction are largely conserved in models generated of SidJ bound to SidE, SdeB, and SdeC, indicating that SidJ is in principle capable of inhibiting all SidE class of enzymes (Supplementary Fig. 11). Accordingly, a previous study showed that SidJ can glutamylate all SidE family members^19^. Interestingly, the ubiquitin-binding surface on the SdeA mART domain overlaps with the SidJ-binding surface, indicating that ubiquitin and SidJ compete for the same surface for binding to SdeA^26^ (Supplementary Fig. 12). The pre-glutamylation structure has no nucleotide bound in the canonical pocket of SidJ, but shows the adenylylated E860 of the SdeA mART domain inserting itself into the migrated pocket of SidJ primed for glutamylation. In the post-catalytic structure of SidJ/CaM-SdeA complex where one cycle of glutamylation occurred, SidJ exists in an apoenzyme form, with no nucleotide bound in either canonical or migrated pockets (Fig. 4). Importantly, part of the loop building the migrated pocket (residues 492–501) becomes disordered after a cycle of glutamylation (Fig. 4C). This observation argues against the proposed allosteric role for the migrated pocket^18^ because the canonical pocket is fully formed even though the migrated pocket is not bound to any nucleotide and disordered in the post-catalytic SidJ/CaM-SdeA structure. Thus, the structures of SidJ/CaM-SdeA presented in this study provide a basis for substrate recognition of SidJ and show that the migrated pocket of SidJ is a dynamic catalytic center that binds to the reaction intermediate-adenylylated SdeA and performs glutamylation.

SidJ autoAMPylation occurs on lysine in the canonical pocket (K370) and also likely on several glutamates in the bridging peptide that connects the canonical pocket and the migrated pocket (Fig. 5, Supplementary Fig. 7, and Supplementary Fig. 8). Mutation of modified glutamate residues in SidJ reduces autoAMPylation but does not affect glutamylation or adenylylation of SdeA, indicating that autoAMPylation events occurring on the bridging peptide are possibly either side reactions of SidJ catalysis or have some unknown role in self-regulation which is yet to be explored. Mutation of K370 completely abolishes the autoAMPylation of SidJ, indicating that modification of K370 precedes that of glutamates in the bridging peptide of SidJ. Compared to WT SidJ, SidJ K370A mutant protein shows strongly reduced glutamylation and adenylylation of SdeA (Fig 5E, F). We also showed that the reduced catalytic activity of the SidJ K370A mutant is not due to a defect in ATP hydrolysis (Fig. 5G). Based on these data, we propose a tentative reaction scheme for the catalysis of SidJ, where the autoAMPylation of SidJ on residue K370 acts as an intermediate in the reaction of SdeA adenylylation and subsequent glutamylation (Supplementary Fig. 13). However, it is important to note that the SidJ K370A mutant protein still showed residual glutamylation and adenylylation activity (Fig. 5D, E). It is unclear how SidJ recognizes SdeA E860 for adenylyation and if/how SidJ K370 autoAMPylation drives this. Further studies are necessary to understand the role of the transient autoAMPylation sites in SidJ catalysis. The structure of the SidJ/CaM-SdeA complex primed for the SdeA adenylylaiton reaction would shed light on the role of K370 and autoAMPylation of SidJ in SidJ-mediate glutamylation of SdeA. It is worth mentioning that DNA ligases carry out lysine AMPylation as an intermediate reaction before AMP is transferred to a DNA 5′ phosphate group and is eventually released upon DNA ligation^28,29^. Additionally, selenoprotein-O (SelO), a conserved pseudokinase, was recently shown to AMPylate substrate proteins as well, but unlike SidJ which catalyzes transient AMPylation of lysine and glutamate residues, SelO targets its substrates on serine, threonine, and tyrosine residues resulting in phosphoester-linked AMPylation of proteins^30^.

SidJ R500A mutant protein exhibits more autoAMPylation and acyl adenylylate formation compared to WT SidJ (Supplementary Fig. 6B). Interestingly, a recent paper by Osinski et al. shows that SidJ R500 coordinates free l-glutamate along with R522 which plays a crucial part in SidJ-enabled nucleophilic attack by l-glutamate^31^. This explains why SidJ R500A mutant shows increased autoAMPylation while being defective in glutamylation of SdeA.

Mammalian glutamylases belong to a single class of tubulin tyrosine ligase-like (TTLL) enzymes that adopt an ATP-grasp fold^32,33^. TTLL enzymes catalyze polyglutamylation of tubulins and play an important role in neuronal development^34^. Unlike SidJ, TTLLs do not adopt a kinase-like fold and possess only one nucleotide-binding site. Despite the differences, TTLLs and SidJ catalyze glutamylation through a similar two-step reaction involving an acyl-phosphate intermediate^19,35^. In the case of TTLLs, the reaction intermediate involves ATP-driven phosphorylation of the target glutamate and in SidJ, it involves AMPylation.

SidJ belongs to a very small number of *Legionella* effectors whose deletion results in a significant reduction in bacterial intracellular growth^11,14,15^. SidJ facilitates *Legionella* replication likely by curbing the excessive toxicity of SidEs which is exerted by their ubiquitination and ubiquitin modification activities^6–8,11,12^. Compared to deletion of SidEs, deletion of SidJ, in general, carries a greater effect on the intracellular replication of *Legionella* in both murine macrophages and amoeba^11^. This could be due to two reasons that are not mutually exclusive: first, that SidJ targets other host cellular substrates during *Legionella* infection, and secondly, prolonged persistence of toxic SidEs in the host cytoplasm due to lack of SidJ is more detrimental to *Legionella*’s replication than the complete lack of SidEs. Future *Legionella* infection experiments complementing a *ΔSidE Legionella* strain with SidE mutants resistant to SidJ, but possessing wild-type activity might help delineate the pathophysiological role of SidJ beyond SidEs.

## Methods

### Purification of SidJ/CaM and SdeA proteins

Constructs of SidJ 99-C or SdeA 231-1190 cloned into pCoofy1 and FL CaM cloned into pET15c were previously published^17,24^. The validated constructs were transformed into chemically competent BL21 Star (Sigma Aldrich) using a heat-shock method, with SidJ being co-expressed with Calmodulin, and SdeA being expressed alone. The cells were grown in LB at 37 °C until OD600 = 0.6, induced using 0.5 mM IPTG, and expressed for 18 h at 18 °C and harvested. The resulting pellets were resuspended in lysis buffer (300 mM NaCl, 50 mM Tris pH 7.5, 10% glycerol) containing the protease inhibitor cocktail (Roche), and lysed using sonication. The lysed cells were then centrifuged at 10,000x*g*, filtered using a 0.22 µm filter, and applied to 3 mL of Talon bead resin (Takara) that was equilibrated into lysis buffer. The clarified lysate was incubated under gentle agitation at 4 °C for 60 min, and centrifuged at 500x*g* for 2 min. The supernatant was decanted, and the beads were washed and incubated for 10 min with lysis buffer three times, each time centrifuging and decanting the supernatant. Further impurities were removed through the addition and incubation of lysis buffer with 10 mM imidazole. The protein was then eluted in multiple steps using imidazole concentrations of 50 to 300 mM, and purity was determined using SDS-PAGE. The pure fractions were concentrated, and loaded onto a pre-equilibrated (100 mM NaCl, 10 mM HEPES pH 7.5, and 0.5 mM TCEP) Superdex S200 increase 10/300 size-exclusion column (GE Life Sciences). Peak fractions were evaluated via SDS-PAGE and the purest fractions were pooled, concentrated to 1.5 mg/mL, and aliquots flash-frozen.

Point mutations were introduced via site-directed mutagenesis using a custom primer pair (as described in Supplementary Table 2).

### Preparation of trapped SidJ/CaM-SdeA heterotrimer for cryo-EM (GraFix)

The sample was trapped and cross-linked using the GraFix protocol^23^. Equimolar quantities of SidJ E565A/CaM and SdeA were incubated on ice for 30 min in the presence of 150 mM NaCl, 50 mM HEPES, 10 mM MgCl2, and 10 mM ATP and loaded onto 5–20% glycerol gradients using 0–0.1% glutaraldehyde and 100 mM NaCl, 10 mM HEPES pH 7.5, 10 mM MgCl2, and the tubes were centrifuged at 164,000x*g* for 18 h at 4 °C. The gradients were subsequently fractionated and quenched through the addition of 10 mM Tris (final concentration). The fractions were evaluated by SDS-PAGE and silver staining using a SilverQuest Silver Stain Kit (Invitrogen). The fractions containing a band of the desired weight were pooled, concentrated, and loaded onto a pre-equilibrated (150 mM NaCl, 10 mM HEPES pH 7.5, 10 mM MgCl2, 0.5 mM TCEP) Superdex S200 3.2/300 size-exclusion column (GE Life Sciences). The peak fractions were pooled and concentrated to 0.3–0.5 mg/mL and used for grid preparation.

### Vitrification and cryo-EM

Two microliters of the cross-linked and concentrated sample were applied using a Vitrobot MkIV (Thermo Scientific) to each side of a Quantifoil Au 300 1.2/1.3 grid, which was glow discharged using a Pelco EasyGlow on both sides using 30 mA for 30 s before being blotted using 100% humidity at 4 °C and blot force −4 or −6 for 2 s and being plunge frozen in liquid ethane. In the case of the post-catalytic structure, 5 mM of Glutamate (final concentration) were added to the sample immediately prior to application to the grid.

The grids were screened using a Glacios 200 kV microscope (Thermo Fisher) with a Falcon III direct electron detector (Thermo Fisher). Movies for the post-catalytic complex were collected in electron counting mode (defocus range of −1.0 to −2.5 µm, 0.25 µm step size) with a magnified pixel size of 0.941 Å at a dose rate of 0.93 e/Å^2^/s for a total dose of 35 e/Å^2^ fractioned over 30 movie frames. Using the same parameters, a short data collection was performed on a grid containing the pre-glutamylation complex, in order to generate an ab initio model for data processing.

For the pre-glutamylation SidJ/CaM-SdeA complex, the movies were collected on a Titan Krios (FEI) equipped with a Quantum-K3 detector (Gatan), a defocus between −0.7 and −1.7 µm was applied in 0.1 µm steps during collection in electron counting mode. A magnified pixel size of 0.504 Å, and a dose rate of 15 e/pix/s for a total dose of 47.45 e/Å^2^ were used, distributed over 40 frames.

### Cryo-EM data processing

To generate an initial model for the catalytic intermediate complex, movies collected on the Glacios 200 kV microscope (Thermo Fisher) were used for particle picking in WARP using the BoxNet2Mask_20180918 model and imported into Relion 3.1^36^ for ab initio 3D classification. The best class was chosen and scaled to serve as the reference model for the high-resolution structure.

For the pre-glutamylation complex, motion correction and contrast-transfer function (CTF) estimation, with subsequent particle picking using the BoxNet2Mask_20180918 model were performed in WARP^37^. Coordinates of 2,683,400 particles were imported into Relion 3.1 and initially extracted with an eightfold binning factor. After one round of reference-free 2D classification, 2,364,848 particles were included for 3D classification, with the previous low-resolution map as a reference. A subset of 432,796 particles from the best two classes were re-extracted with a binning factor of 4, classified again, and 140,022 particles from the best class were picked and refined to 4.15 Å resolution. The particles were once again re-extracted using a binning factor of 2, and refined to a resolution of 3.36 Å, before CTF refinement, beam-tilt correction, and Bayesian particle polishing were performed. These steps resulted in a model resolution of 2.94 Å. The reported overall resolution of 2.9 Å was calculated using the gold-standard Fourier shell correlation (FSC) 0.143 criterion^38^ and was corrected for the effects of a soft mask on the FSC curve using high-resolution noise substitution^39^.

For the post-catalytic complex, motion correction and contrast-transfer function (CTF) estimation, with subsequent particle picking using the BoxNet2Mask_20180918 model were performed in WARP. Coordinates of 635,561 particles were imported into Relion 3.1 and initially extracted with a fourfold binning factor. All imported particles were included for 3D classification to generate an ab initio model. A subset of 94,463 particles from the best class were refined to 7.72 Å resolution. The particles were re-extracted without binning, and refined to a resolution of 3.81 Å, before CTF refinement, beam-tilt correction, and Bayesian particle polishing were performed. After another round of 3D refinement, which produced a model of 3.76 Å resolution, the particles were classified one more time, and 58,448 particles from the best class were picked for a final round of 3D refinement. These steps resulted in a map resolution of 3.71 Å.

Models were built using PDB models for SidJ/CaM (PDB 6OQQ) and SdeA (5YIM) as starting points and further refined using Coot 0.9.5 and Phenix 1.14.

### Immunoprecipitation

HEK293 cells were co-transfected transiently with mCherry-SidJ (pmCherry-C1 vector) and either GFP (pEGFP-C1 vector) or GFP-SdeA (pEGFP-C1) using polyethylenimine. Cells were harvested at 18 h post-transfection, washed with PBS (phosphate-buffered saline), and lysed in immunoprecipitation buffer (50 mM Tris-HCl pH 7.5, 150 mM NaCl, 1% Triton X-100, and protease inhibitor cocktail (cOmplete Mini EDTA-free from Roche) and mixed with GFP-Trap Agarose beads (ChromoTek) and incubated for 2 h at 4 °C while being subjected to end-to-end rotation. The beads were washed three times with the immunoprecipitation buffer. Finally, proteins were eluted by boiling with 4x Laemmli buffer for 10 min, then separated through SDS-PAGE, and visualized following western blot. The Antibody used for mCherry detection was DSRed2 sc-101256 with a dilution of 1:1000 (Santa Cruz Biotechnology), and GFP was detected with GFP sc-9996 and a dilution of 1:2000 (Santa Cruz Biotechnology). The raw western blot image is available in the supplementary information file.

### Glutamylation assay

About 20 uL in vitro glutamylation reactions were performed with 2uM SidJ/CaM and 2uM SdeA, 5 mM ATP, and 50 uM (0.5 nCi) l-[14 C]glutamate (Perkin Elmer) in a buffer consisting of 30 mM HEPES pH 7.5, 150 mM NaCl, 10 mM MgCl2, 1 mM β-mercaptoethanol, and incubated at 37 °C for 30 min. Reactions were stopped through the addition of 5 uL of 4x SDS-PAGE sample buffer. The samples were then separated using SDS-PAGE, stained using Coomassie stain, and dried using a Model 583 gel dryer (Bio-Rad). A storage phosphor screen (GE Healthcare) was placed on the gel and exposed for 72 h, and the autoradiography signal was collected using a Typhoon FLA 7000 (GE). Raw autoradiography images of all glutamylation assays are available in the supplementary information file.

### Acyl adenylate formation assay

AMPylation was measured using the protocol described in Black et al. (2019). Briefly, reactions were carried out in the presence of 150 µM (5 µCi) [α-32P]ATP. The 20 µL reactions contained 1 mg/ml bovine serum albumin (BSA), 100 mM sodium acetate, 150 mM NaCl, 50 mM Tris, 50 mM Bis-Tris pH 6.5, 0.5 mM MgCl2, and 1 mM DTT, and equimolar quantities of 5 µM SidJ/CaM and SdeA. Reactions were carried out on ice and stopped after 30 min by adding 500 µL of ice-cold 20% TCA and incubated on ice for 40 min. Products were centrifuged at 21,000x*g* for 15 min, before washing the pellet twice with 250 µL of ice-cold TCA. The radioactivity of the acid-insoluble pellet was measured through the addition of 50 µL of MicroScint PS (Perkin Elmer), vortexing, and performing scintillation counting on a MicroBeta 2450 Microplate Counter (Perkin Elmer). The graphs were prepared using GraphPad Prism. Raw data of the acyl adenylate formation assay are supplied in the source data table.

### AutoAMPylation assay

About 20 µL in vitro glutamylation reactions were performed with 2 µM SidJ/CaM, 2.5µCi α -[32 P]ATP (Perkin Elmer) in a buffer consisting of 30 mM HEPES pH 7.5, 150 mM NaCl, 10 mM MgCl2, 1 mM β-mercaptoethanol, and incubated at 37 °C for 30 min. Reactions were stopped through the addition of 5 µL of 4x SDS-PAGE sample buffer. The samples were then separated using SDS-PAGE, stained using Coomassie stain, and dried using a Model 583 gel dryer (Bio-Rad). A storage phosphor screen (GE Healthcare) was placed on the gel and exposed for 18 h, and the autoradiography signal was collected using a Typhoon FLA 7000 (GE). Raw autoradiography images of all autoAMPylation assays are available in the supplementary information file.

### Analytical gel filtration

Analytical gel filtrations were performed using equimolar mixtures of SidJ/CaM constructs and SdeA constructs at a concentration of 0.5 mg/mL. Under the addition of 10 mM MgCl and 5 mM ATP, the sample was incubated on ice for 30 min before being loaded onto a pre-equilibrated (100 mM NaCl, 10 mM HEPES pH 7.5, 100 mM NaCl, 10 mM MgCl2, and 0.5 mM TCEP) Superdex S200 3.2/300 size-exclusion column (GE Life Sciences). The resulting chromatograms were overlaid and compared to calibration curves to estimate molecular weight. Chromatograms were plotted using GraphPad Prism. Raw data of all chromatograms shown are available in the source data file.

### Mass spectrometry

Reaction mixtures were digested through the addition of 500 ng of Promega Trypsin Gold (resuspended in 500 nl of 50 mM acetic acid) and 20 ul 50 mM ammonium bicarbonate (ABC). After 90 min incubation at room temperature digests were applied to in-house manufactured C18 StageTips equilibrated with 10 mM ABC. Samples were washed with 10 mM ABC, eluted with 40% acetonitrile in 10 mM ABC and dried for 1 h by vacuum centrifugation.

LC–MS analyses were performed using either an Easy-nLC 1000 coupled to an Orbitrap Fusion mass spectrometer or an Easy-nLC 1200 coupled to an Orbitrap Fusion Lumos (Thermo Scientific) with peptides generated from roughly 500 ng of proteins injected for each analysis onto either 75 microns × 25 cm packed emitter columns (New Objective) or 75 microns × 50 cm C18 Acclaim Pepmap columns (Thermo Scientific). Peptides were eluted with linear gradients from 1 to 35% solvent B (80% acetonitrile in 0.1% formic acid) in either 30 or 45 min, followed by a steeper wash phase.

In general, samples were analyzed with “Topspeed” data-directed analysis methods with a cycle time setting of 2 or 3 s. In order to obtain high-quality fragmentation data, the MS2 AGC fill was set high for both HCD (300–500%) and ETD (200%) acquisitions. In some acquisitions, rapid HCD fragmentation was performed with standard parameters to screen for AMPylated peptides. The presence of adenine (ADN) fragment ion (136,062 Da) was used to trigger higher-quality HCD or ETD fragmentation. c and z ions are a result of electron transfer dissociation (ETD) fragmentation which fragments the peptide backbone at the N-C_α_ bond.

Due to the possible liability of the AMP modification, various acquisition strategies were applied. Higher-energy collision-induced decay (HCD) was applied initially in order to identify modified peptides. This mode of fragmentation tends to break off the modifier. Interestingly two fragmentation behaviors were observable. Diagnostic ions for breakage of AMP appeared at either 330,060 (representing the broken-off AMP modifier itself in the H+ state) or 348,071 (AMP having taken an additional H2O from the side chain). In the peptides modified on lysine, the AMP + H2O ion does not appear, presumably since there is no oxygen involved in binding to the lysine side chain. In those peptides where the modifier appears to be on E, the heavier diagnostic ion is observed. We would propose that this heavier diagnostic ion is characteristic of O-linked AMPylation.

### Pyrophosphate release assay

The ATPase activity of WT SidJ and its mutants was measured in a UV-transparent microplate using the EnzChek Pyrophosphate Assay Kit (Thermo Fisher Scientific, E-6645). The assay was performed in triplicates. All the components were added to the reaction mixture as described in the kit. About 0.5 µM of WT SidJ, and mutants were added into the reaction mixture. Finally, 2 mM ATP was then added into the reaction mixture to start the reaction and absorbance measurements at 360 nm were taken immediately and continuously at 1-min intervals using a Clariostar plate reader. Source data taken during the experiment are available in the source data file.

### Statistics and reproducibility

The assays shown in Figs. 1a, e, 2d, 3d, f, 4b, 5a, d, e, f, g, Supplementary Figs. 1a, 1f, and 6a, b, c of this publication have been performed at least three independent times with similar results.

### Reporting Summary

Further information on research design is available in the Nature Research Reporting Summary linked to this article.

## Supplementary information

Supplementary Information

Peer Review File

Reporting summary

## Data Availability

Mass spectrometry data are available from the Proteomics Identification (PRIDE) database with the dataset identifier PXD028638. Cryo-EM structure coordinates are available from the Protein Data Bank (PDB) and the Electron Microscopy Data Bank (EMDB) for the catalytic intermediate under accession codes 7PPO and EMD-13583, respectively. The coordinates and cryo-EM density for the post-catalytic complex are available using accession codes 7PQE and EMD-13591. Source data are provided with this paper.

## Source data

Source Data

## References

[1] Grabbe C, Husnjak K, Dikic I (2011). The spatial and temporal organization of ubiquitin networks. Nat. Rev. Mol. Cell Biol..

[2] Yau R, Rape M (2016). The increasing complexity of the ubiquitin code. Nat. Cell Biol..

[3] Maculins T, Fiskin E, Bhogaraju S, Dikic I (2016). Bacteria-host relationship: ubiquitin ligases as weapons of invasion. Cell Res..

[4] Hubber, A. & Roy, C. R. Modulation of host cell function by *Legionella* pneumophila type IV effectors. **26**, 261–283 (2010). 10.1146/annurev-cellbio-100109-104034 20929312

[5] Xu L, Luo Z-Q (2013). Cell biology of infection by *Legionella pneumophila*. Microbes Infect..

[6] Qiu J (2016). Ubiquitination independent of E1 and E2 enzymes by bacterial effectors. Nature.

[7] Bhogaraju S (2016). Phosphoribosylation of ubiquitin promotes serine ubiquitination and impairs conventional ubiquitination. Cell.

[8] Kotewicz KM (2017). A single *Legionella* effector catalyzes a multistep ubiquitination pathway to rearrange tubular endoplasmic eeticulum for replication. Cell Host Microbe.

[9] Zhang, M. et al. Members of the *Legionella pneumophila* Sde family target tyrosine residues for phosphoribosyl-linked ubiquitination. *RSC Chem. Biol.* **2**, 1509–1519 10.1039/D1CB00088H (2021). 10.1039/d1cb00088h PMC8496037 34704056

[10] Urbanus ML (2016). Diverse mechanisms of metaeffector activity in an intracellular bacterial pathogen, *Legionella pneumophila*. Mol. Syst. Biol..

[11] Jeong KC, Sexton JA, Vogel JP (2015). Spatiotemporal regulation of a *Legionella pneumophila* T4SS substrate by the metaeffector SidJ. PLoS Pathog..

[12] Havey JC, Roy CR (2015). Toxicity and SidJ-mediated suppression of toxicity require distinct regions in the SidE family of *Legionella pneumophila* effectors. Infect. Immun..

[13] Qiu J (2017). A unique deubiquitinase that deconjugates phosphoribosyl-linked protein ubiquitination. Cell Res..

[14] Liu Y, Luo Z-Q (2007). The *Legionella pneumophila* effector SidJ is required for efficient recruitment of endoplasmic reticulum proteins to the bacterial phagosome. Infect. Immun..

[15] Bardill JP, Miller JL, Vogel JP (2005). IcmS‐dependent translocation of SdeA into macrophages by the *Legionella pneumophila* type IV secretion system. Mol. Microbiol..

[16] Jeong KC, Sutherland MC, Vogel JP (2015). Novel export control of a *Legionella* Dot/Icm substrate is mediated by dual, independent signal sequences. Mol. Microbiol..

[17] Bhogaraju S (2019). Inhibition of bacterial ubiquitin ligases by SidJ-calmodulin catalysed glutamylation. Nature.

[18] Sulpizio A (2019). Protein polyglutamylation catalyzed by the bacterial calmodulin-dependent pseudokinase SidJ. Elife.

[19] Black MH (2019). Bacterial pseudokinase catalyzes protein polyglutamylation to inhibit the SidE-family ubiquitin ligases. Science.

[20] Shin D (2020). Regulation of phosphoribosyl-linked serine ubiquitination by deubiquitinases DupA and DupB. Mol. Cell.

[21] Wan M (2019). Deubiquitination of phosphoribosyl-ubiquitin conjugates by phosphodiesterase-domain-containing *Legionella* effectors. Proc. Natl Acad. Sci. USA.

[22] Gan N (2019). Regulation of phosphoribosyl ubiquitination by a calmodulin-dependent glutamylase. Nature.

[23] Kastner B (2008). GraFix: sample preparation for single-particle electron cryomicroscopy. Nat. Methods.

[24] Kalayil S (2018). Insights into catalysis and function of phosphoribosyl-linked serine ubiquitination. Nature.

[25] Akturk A (2018). Mechanism of phosphoribosyl-ubiquitination mediated by a single *Legionella* effector. Nature.

[26] Dong Y (2018). Structural basis of ubiquitin modification by the *Legionella* effector SdeA. Nature.

[27] Wang Y (2018). Structural insights into non-canonical ubiquitination catalyzed by SidE. Cell.

[28] Gumport RI, Lehman IR (1971). Structure of the DNA ligase-adenylate intermediate: lysine (ε-amino)-linked adenosine monophosphoramidate. Proc. Natl Acad. Sci. USA.

[29] Itzen A, Blankenfeldt W, Goody RS (2011). Adenylylation: renaissance of a forgotten post-translational modification. Trends Biochem. Sci..

[30] Sreelatha A (2018). Protein AMPylation by an evolutionarily conserved pseudokinase. Cell.

[31] Osinski, A. et al. Structural and mechanistic basis for protein glutamylation by the kinase fold. *Mol. Cell*. 10.1016/j.molcel.2021.08.007 (2021). 10.1016/j.molcel.2021.08.007 PMC8571041 34407442

[32] Janke C (2005). Tubulin polyglutamylase enzymes are members of the TTL domain protein family. Science.

[33] Szyk A, Deaconescu AM, Piszczek G, Roll-Mecak A (2011). Tubulin tyrosine ligase structure reveals adaptation of an ancient fold to bind and modify tubulin. Nat. Struct. Mol. Biol..

[34] Janke C, Magiera MM (2020). The tubulin code and its role in controlling microtubule properties and functions. Nat. Rev. Mol. Cell Biol..

[35] Mahalingan KK (2020). Structural basis for polyglutamate chain initiation and elongation by TTLL family enzymes. Nat. Struct. Mol. Biol..

[36] Scheres SHW (2012). RELION: implementation of a Bayesian approach to cryo-EM structure determination. J. Struct. Biol..

[37] Tegunov D, Cramer P (2019). Real-time cryo-electron microscopy data preprocessing with Warp. Nat. Methods.

[38] Scheres SHW, Chen S (2012). Prevention of overfitting in cryo-EM structure determination. Nat. Methods.

[39] Chen S (2013). High-resolution noise substitution to measure overfitting and validate resolution in 3D structure determination by single particle electron cryomicroscopy. Ultramicroscopy.

