# Supplementary material for: Structural basis for protein glutamylation by the Legionella pseudokinase SidJ
Source: Nat Commun. 2021 Oct 26;12:6174. doi: 10.1038/s41467-021-26429-y (PMC8548325; doi:10.1038/s41467-021-26429-y)
Supplement: Supplementary file 1 — Supplementary Information [file 41467_2021_26429_MOESM1_ESM.pdf]

Supplementary Figure 1 – SidJ - SdeA interaction is transient

A) mCherry-SidJ and either GFP-SdeA or GFP expressed in HEK293T cells. GFP IP was performed followed by blotting with anti-GFP and anti-mCherry antibodies

B) Detailed view of SidJ canonical pocket with mutated residues shown

C) Detailed view of SidJ migrated pocket with mutated residues shown

D) SEC profiles of SidJ/CaM point mutants mixed with SdeA in the presence of ATP

E) SEC profiles of SidJ-E565A/CaM and SdeA with and without ATP, as well as SidJ/CaM and SdeA alone

F) Incorporation of [^{14}C]-Glu into SdeA catalyzed by SidJ using SidJ WT, SidJ H492A, SidJ E565A, and cross-linked SidJE565A/CaM/SdeA at 37°C (Prepared as in Figure 1E). Samples were separated by SDS-PAGE and visualized by Coomassie stain (Bottom) or autoradiography (Top)

Supplementary Figure 1

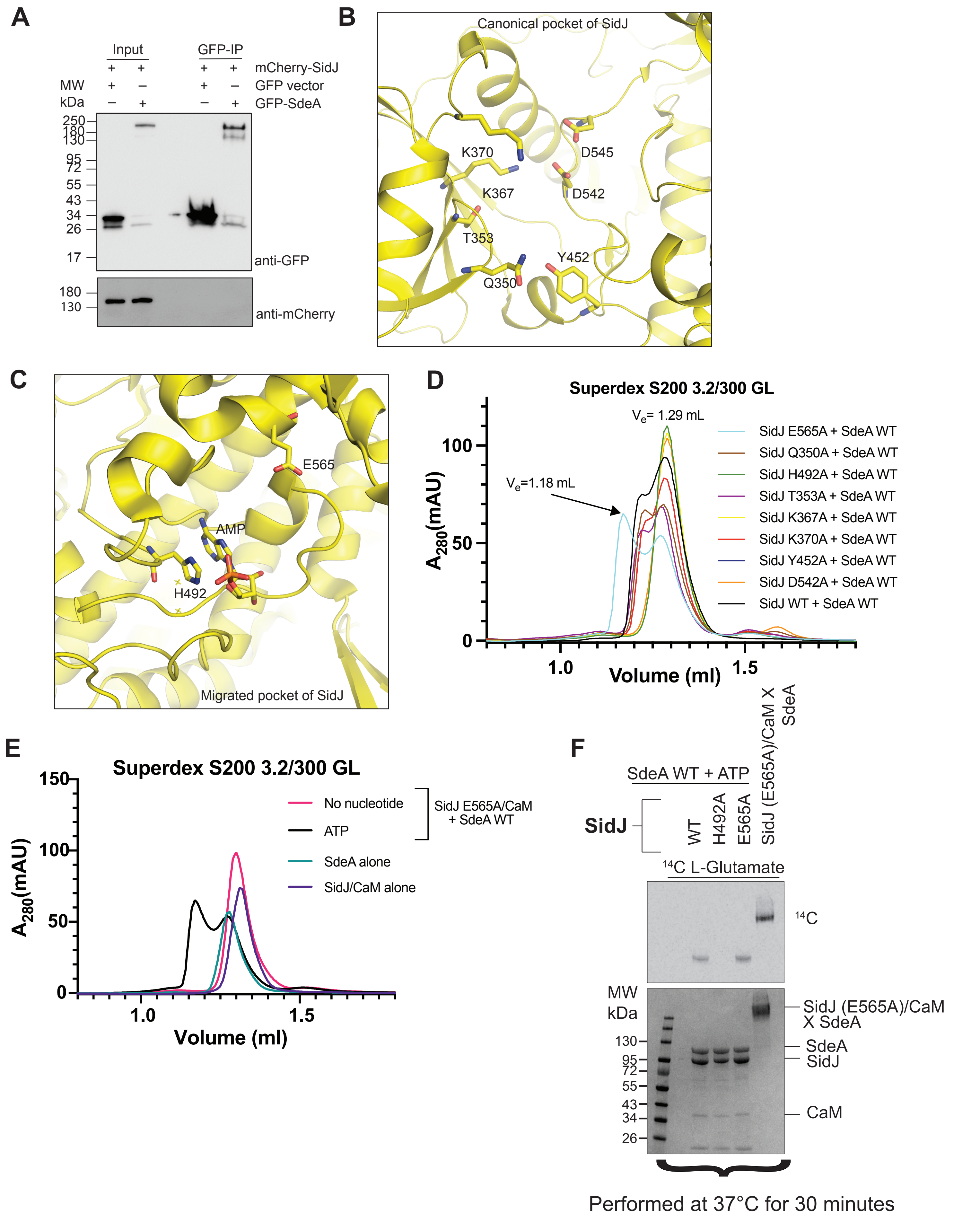

Supplementary Figure 2 – Processing pipeline of SidJE-565A/CaM/SdeA reaction intermediate complex

Particles were picked using WARP and processed using RELION, pipeline shows models up to final 3D refinement step. Bottom right, Gold Standard FSC curves of SidJ-E565A/CaM/SdeA pre-glutamylation complex, and final map of SidJE-565A/CaM/SdeA colored according to the local resolution estimates.

Supplementary Figure 2

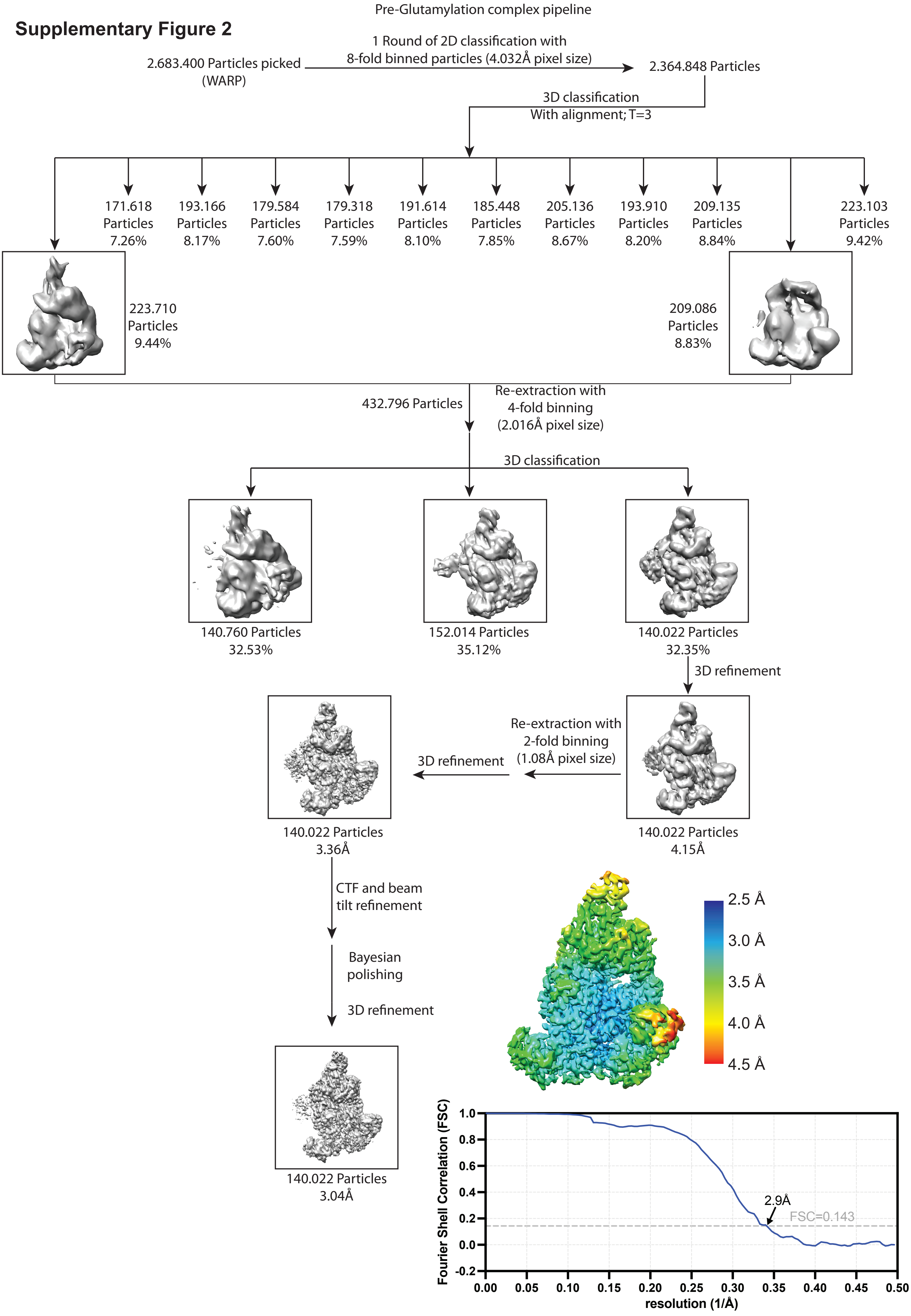

Supplementary Figure 3 – Structural comparison of SidJ and SdeA alone and in complex

A) Comparison between SidJ/CaM crystal structure (Grey, PDB: 6OQQ) and SidJ/CaM in the pre-glutamylaiton complex SidJ-E565A/CaM/SdeA (Yellow, Teal)

B) Comparison between SdeA crystal structure (Grey, PDB: 5YIJ) and SdeA in the the pre-glutamylaiton complex SidJ-E565A/CaM/SdeA (Green)

Supplementary Figure 3

A

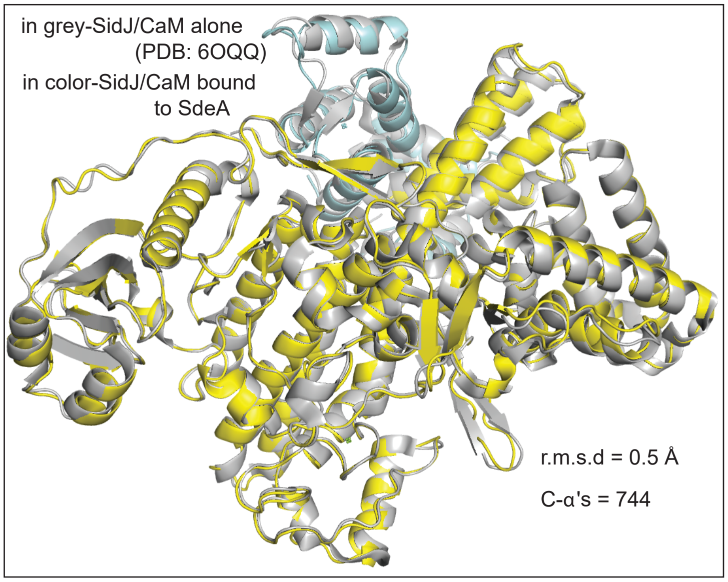

B

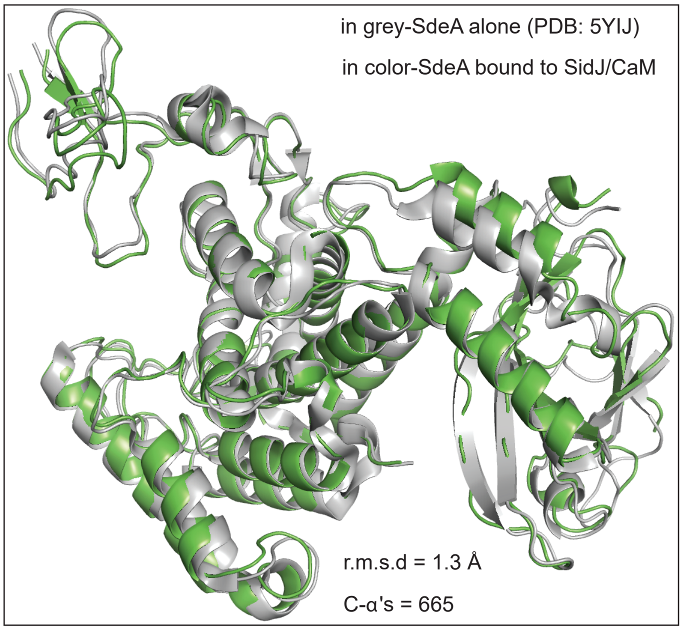

Supplementary Figure 4 – SidJ mutation E565A does not induce structural change

A) Comparison of SidJ/CaM migrated pocket with AMP bound in crystal structure (Grey, PDB: 6OQQ) and SidJ-E565A/CaM/SdeA with AMPylated E860 bound in the catalytic intermediate complex (Colored)

B) Comparison of SidJ/CaM migrated pocket structure in WT-SidJ crystal structure (Grey) and SidJ-E565A/CaM/SdeA catalytic intermediate complex

C) Electrostatic potential is projected onto the surface of SidJ WT (PDB: 6S5T) and SidJ E565A (modeled) migrated pocket. AMP is modeled based on the crystal structure of SidJ (PDB: 6OQQ)

Supplementary Figure 4

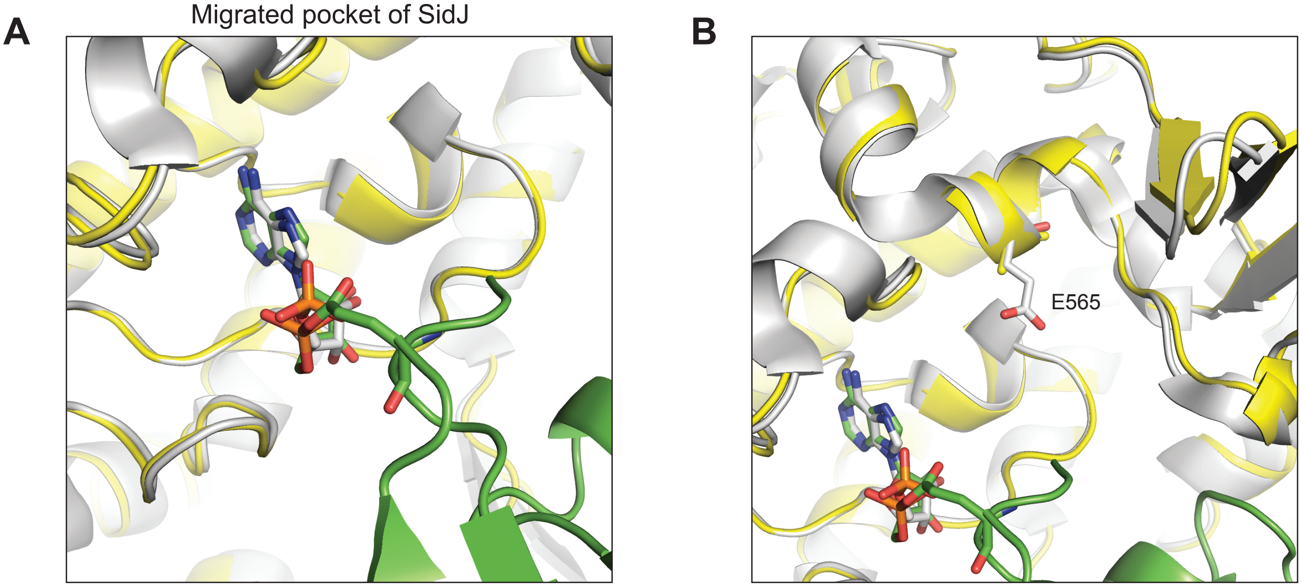

in grey-SidJ/CaM bound to AMP crystal structure (PDB: 6OQQ)

in color-SidJ/CaM bound to Adenylated SdeA

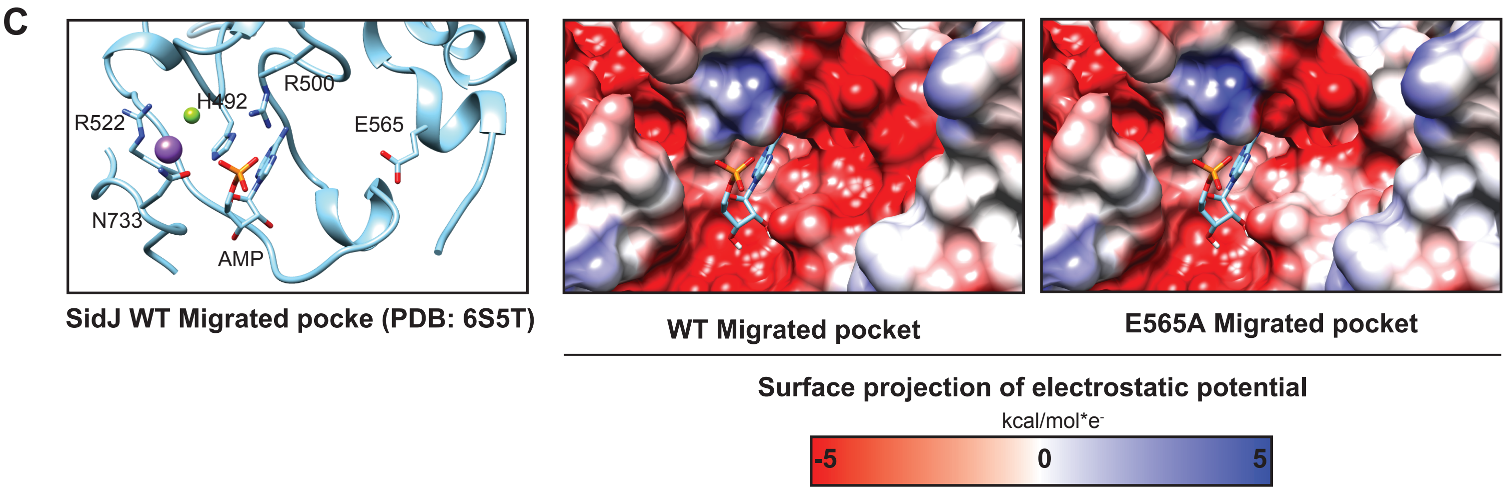

Supplementary Figure 5 - Processing pipeline of SidJE-565A/CaM/SdeA post-catalytic complex

Particles were picked using WARP and processed using RELION, pipeline shows models up to final 3D refinement step. Bottom left, Gold Standard FSC curves of SidJ-E565A/CaM/SdeA post-catalytic complex. Bottom right, Final map of the post-catalytic SidJE-565A/CaM/SdeA complex colored according to the local resolution estimates.

Supplementary Figure 5

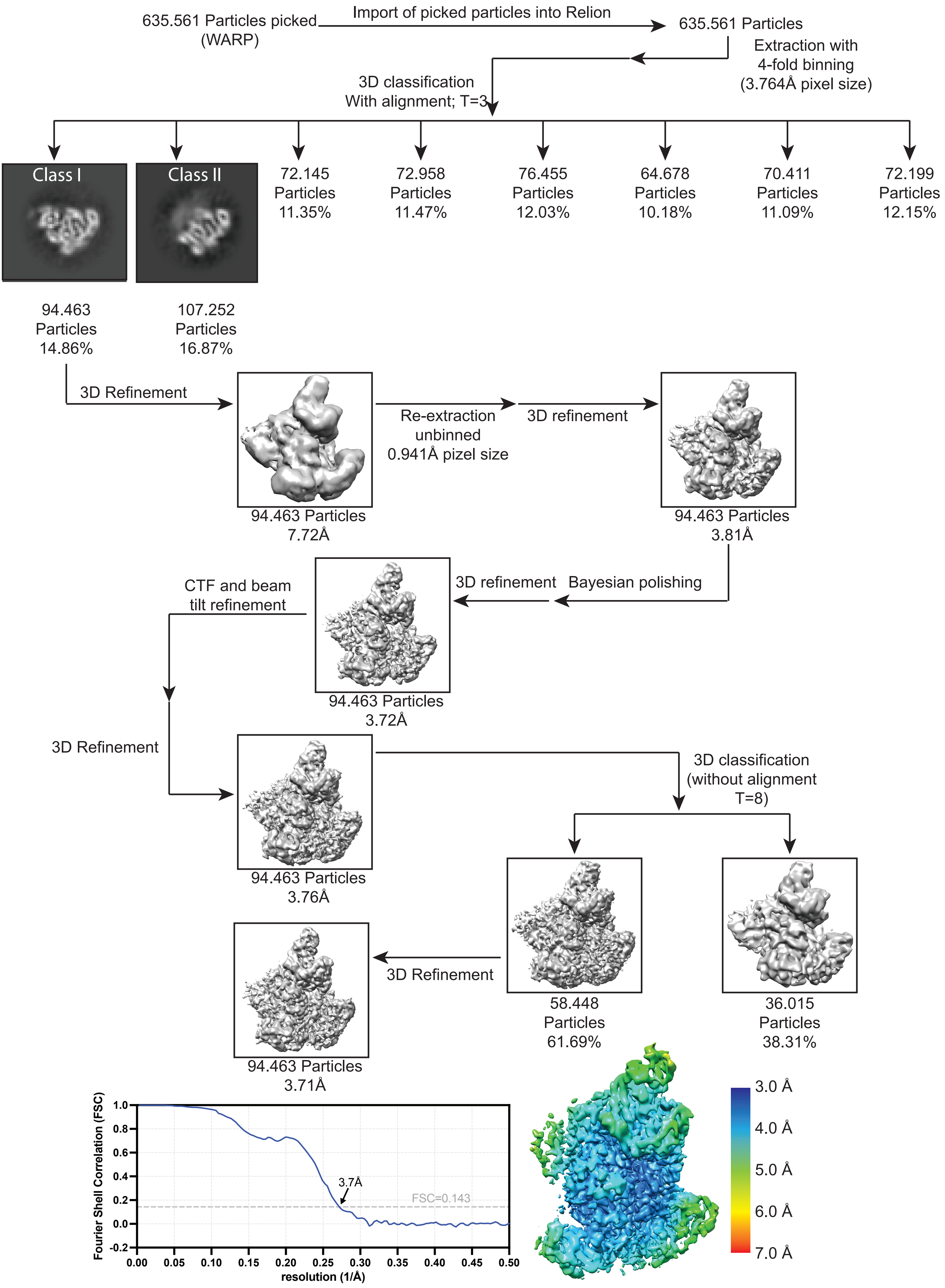

Supplementary Figure 6 – Characterization of SidJ R500A mutant

A) Incorporation of α-[^{32}P]-ATP into various SidJ constructs as indicated. Proteins were incubated with α-[^{32}P]-ATP. Reactions were separated by SDS-PAGE and visualized by autoradiography.

B) Left, incorporation of α-[^{32}P]-ATP into SidJ or SidJ R500A. After the reaction the samples were treated at the indicated temperature for various time points in 0.1% formic acid. Reactions were separated by SDS-PAGE and visualized by autoradiography. Data shown in figure 5b is a cropped version of data shown in this figure. Right, Acyl adenylylate formation after reaction with SidJ WT or mutants in the presence of WT SdeA and α-[32P]-ATP. Reactions were terminated by the addition of TCA, and TCA-insoluble pellets were measured by scintillation counting. Measurements for each sample were taken in triplicates (n=3), error bars denote standard deviation.

Supplementary Figure 6

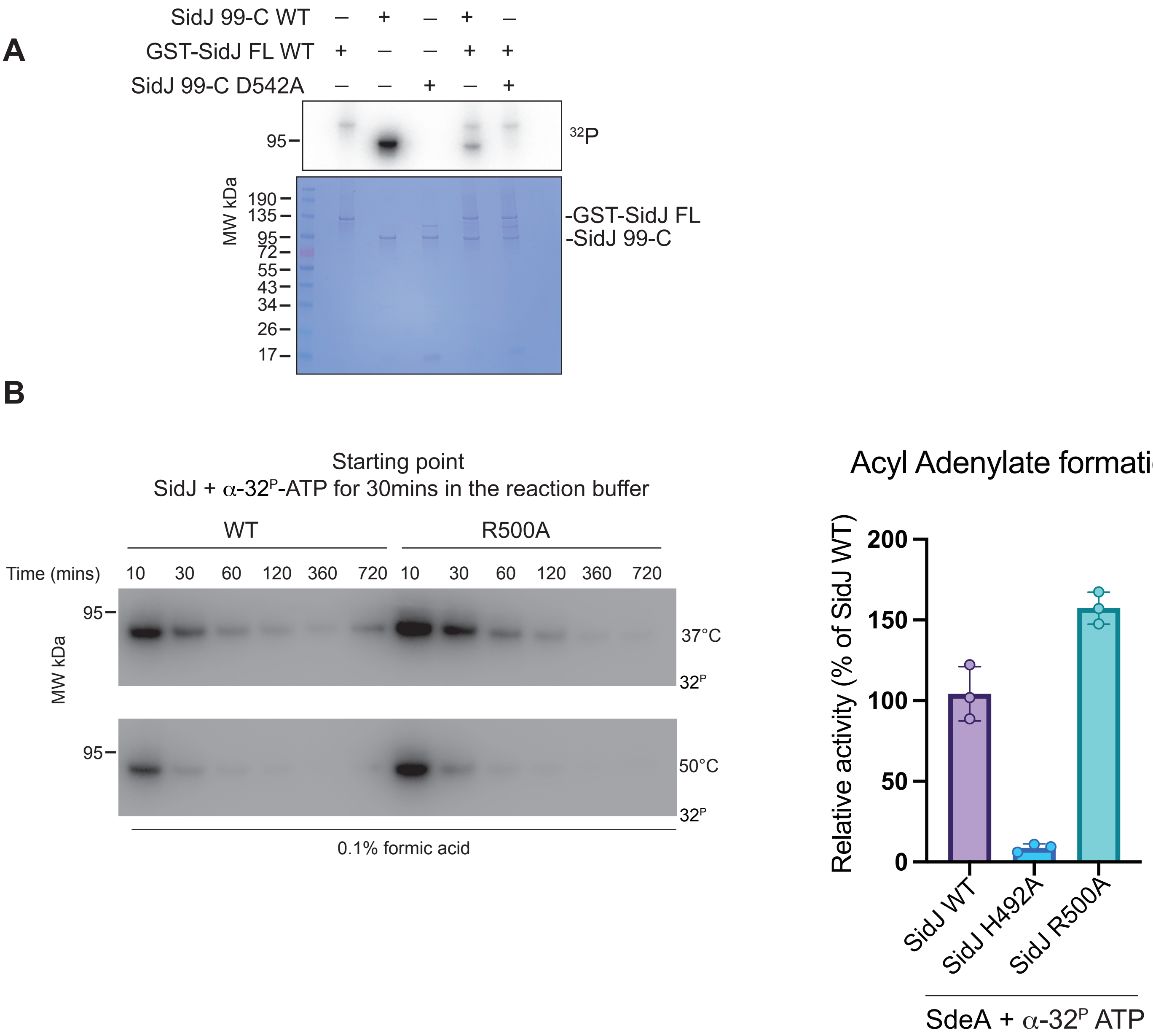

Supplementary Figure 7 – Mass spectrometry analysis of SidJ-autoAMPylation.

A) LC-MS/MS of SidJ R500A autoAMPylation reaction showing the HCD fragmentation spectra of the canonical pocket peptide.

B) LC-MS/MS of SidJ R500A autoAMPylation reaction showing the HCD fragmentation of the bridging peptide.

C) HCD Fragmentation spectrum of SidJ R500A peptide E480-K503 with a possible AMPylation site E499 highlighted

Supplementary Figure 7

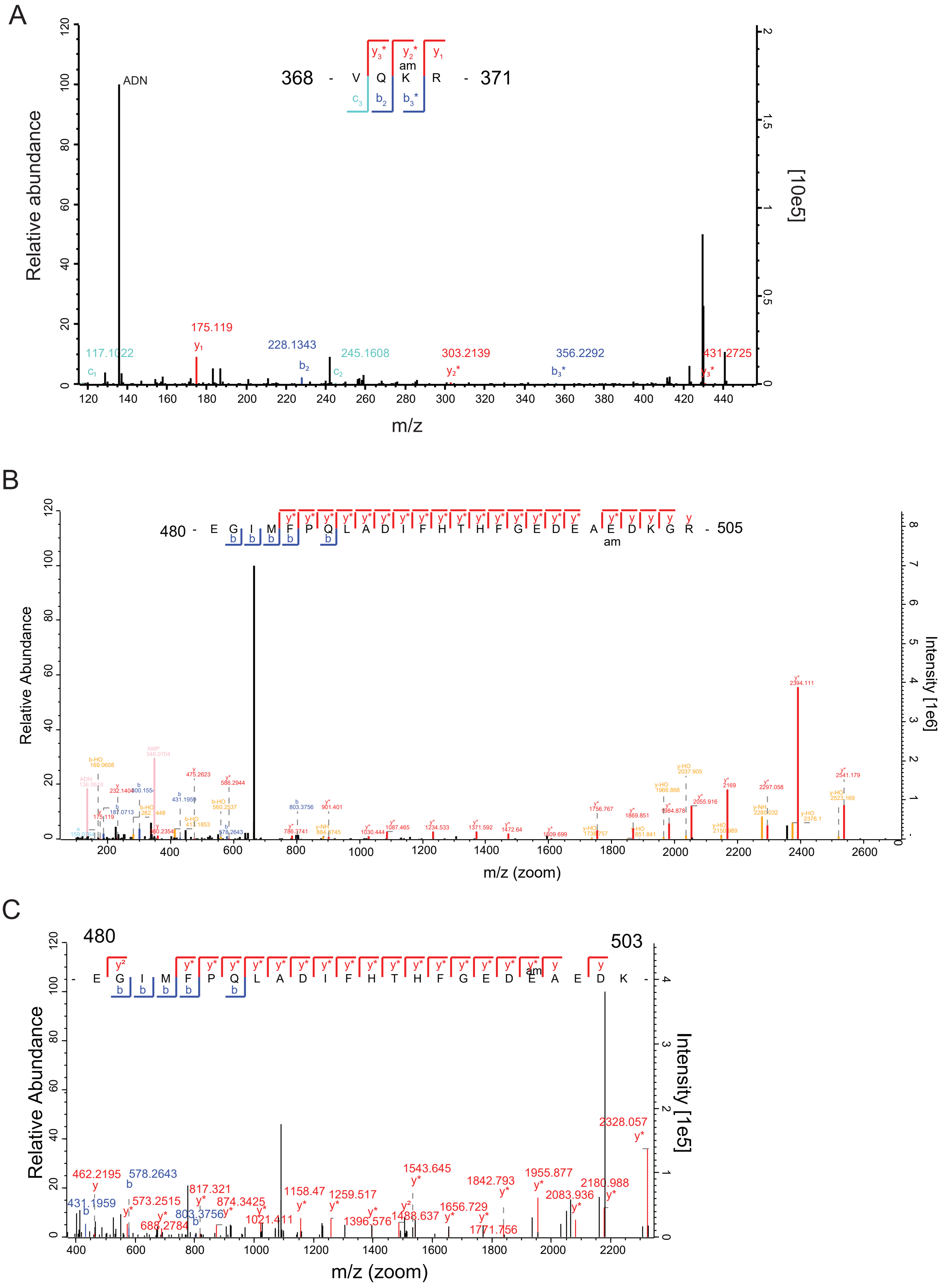

Supplementary Figure 8 – HCD fragmentation spectra showing the AMPylation of SdeA mART catalytic peptide.

A) LC-MS/MS of SidJ WT autoAMPylation reaction showing the ETD Fragmentation of the bridging peptide.

B) HCD fragmentation spectrum of SdeA catalytic peptide. A reaction was set up with SidJ R500A/CaM with SdeA in the presence of ATP. The reaction components were subjected to LC-MS/MS.

Supplementary Figure 8

A

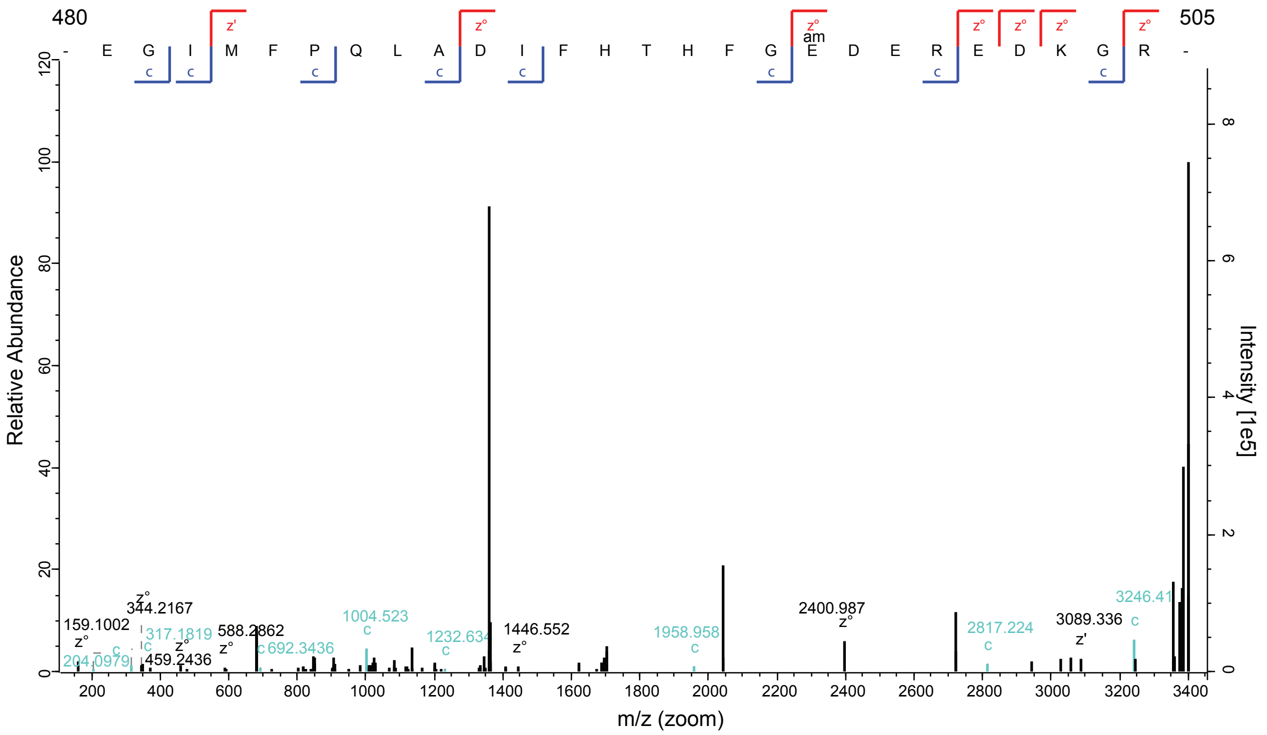

B

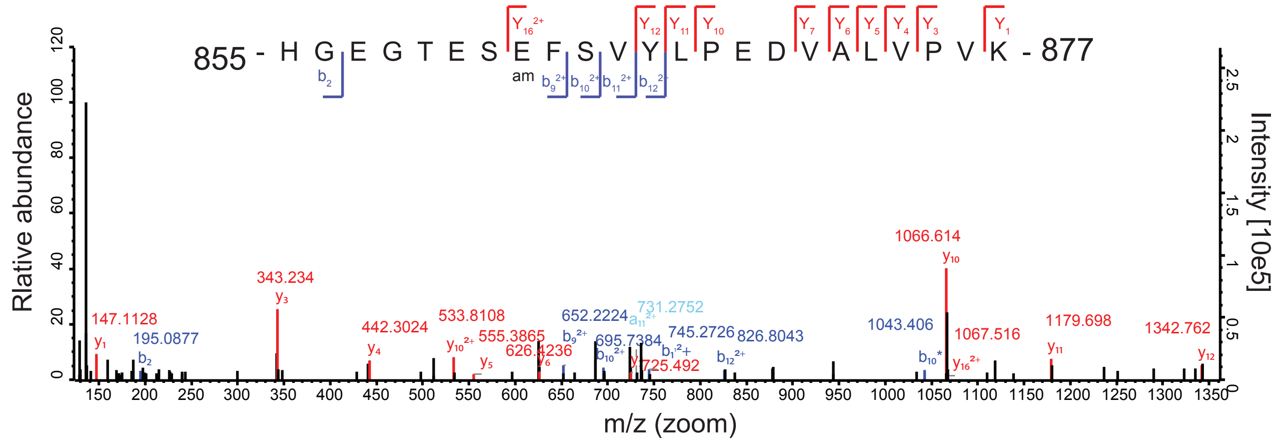

Supplementary Figure 9 – SidJ structure showing the cluster of glutamate residues around the autoAMPylation sites found by mass spectrometry

Detailed view of SidJ auto-AMPylation sites, with peptides recovered in mass spec shown in red, modification sites including a series of glutamates extending into the migrated pocket are highlighted

Supplementary Figure 9

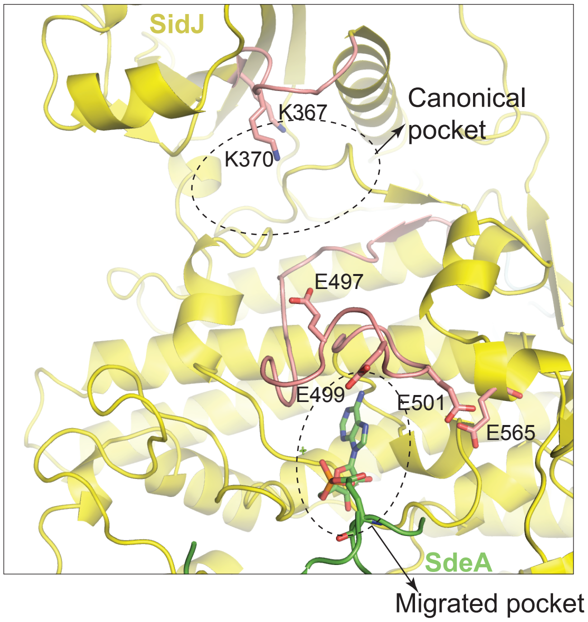

Supplementary Figure 10 – Multiple sequence alignment of SidE family enzymes.

Residues important for binding to SidJ are highlited.

Supplementary Figure 10

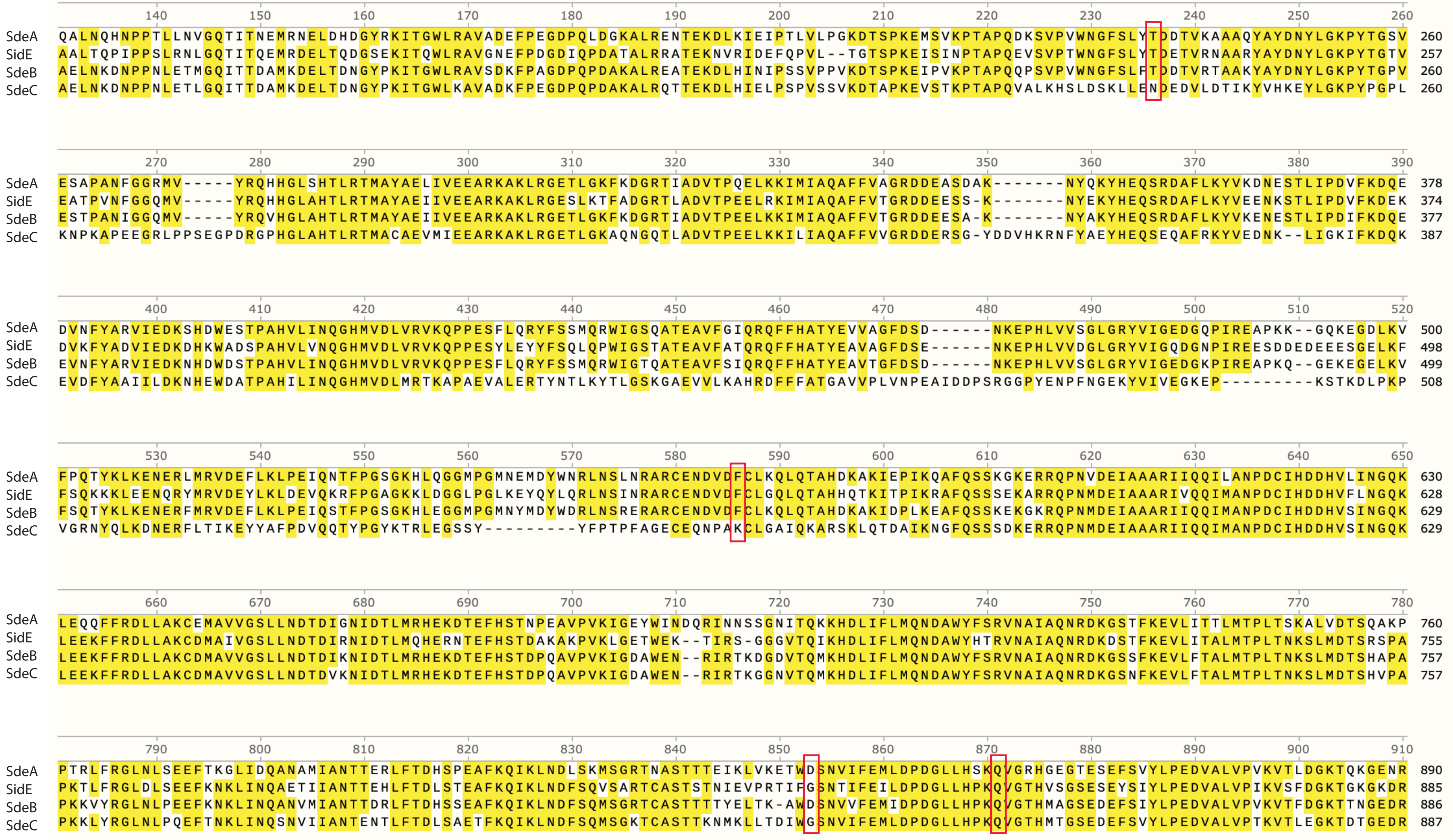

Supplementary Figure 11 – Structural comparison of SidE family members in complex with SidJ

Models for SdeB and SdeC were generated using AlphaFold2, the model of SidE was taken from PDB 5ZQ2. The three binding sites between SidJ and SdeA are defined in 2A. Models of SidE family members were structurally aligned to the intermediate complex of SidJ/CaM-SdeA, and the homologous residues of SidE family members are shown.

Supplementary Figure 11

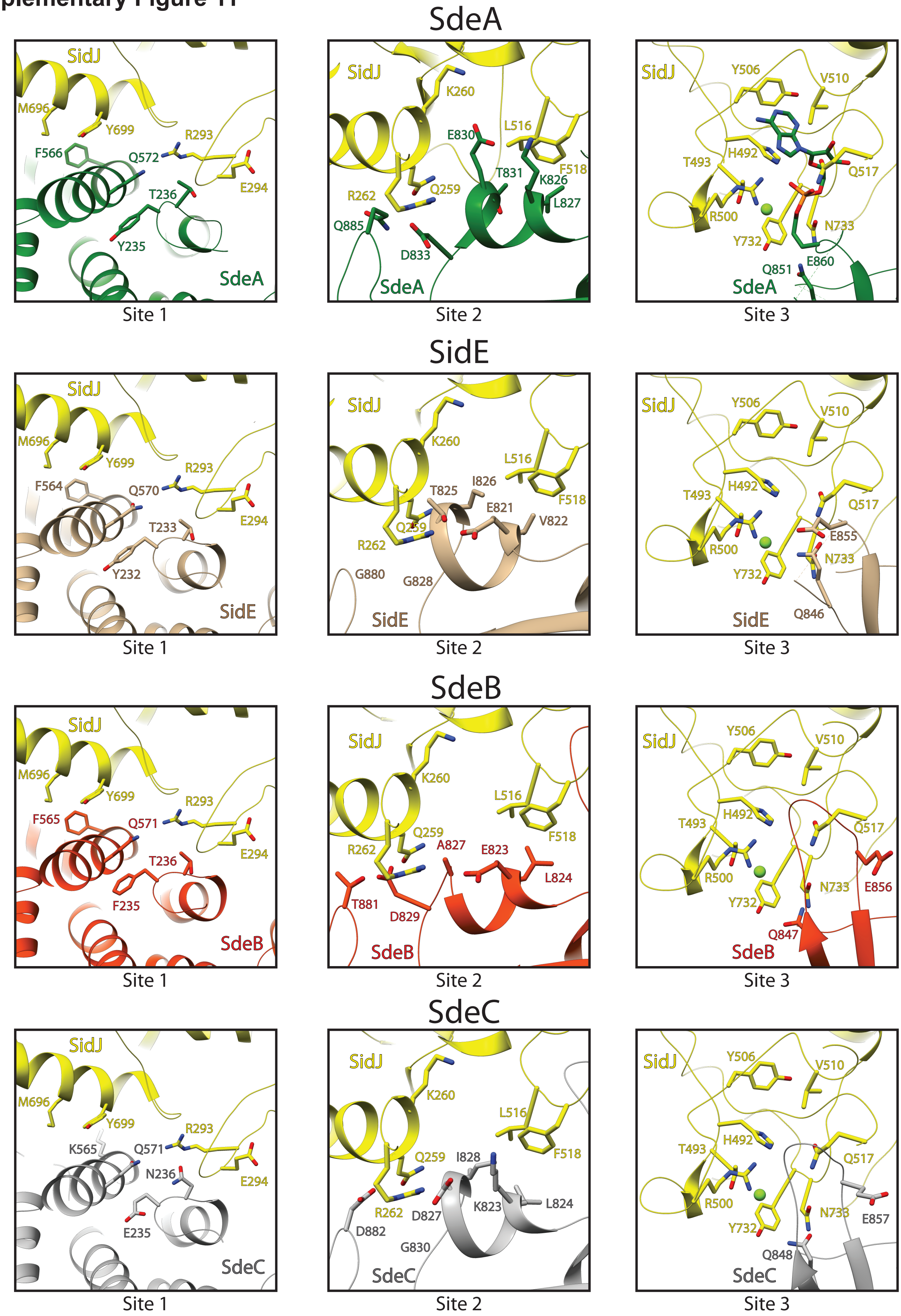

Supplementary Figure 12 – SidJ and Ubiquitin compete for the same binding surface on the mART domain of SdeA

SdeA-Ub complex structure (PDB: 5YIJ) is superposed on the pre-glutamylation complex structure of SidJ/CaM-SdeA.

Supplementary Figure 12

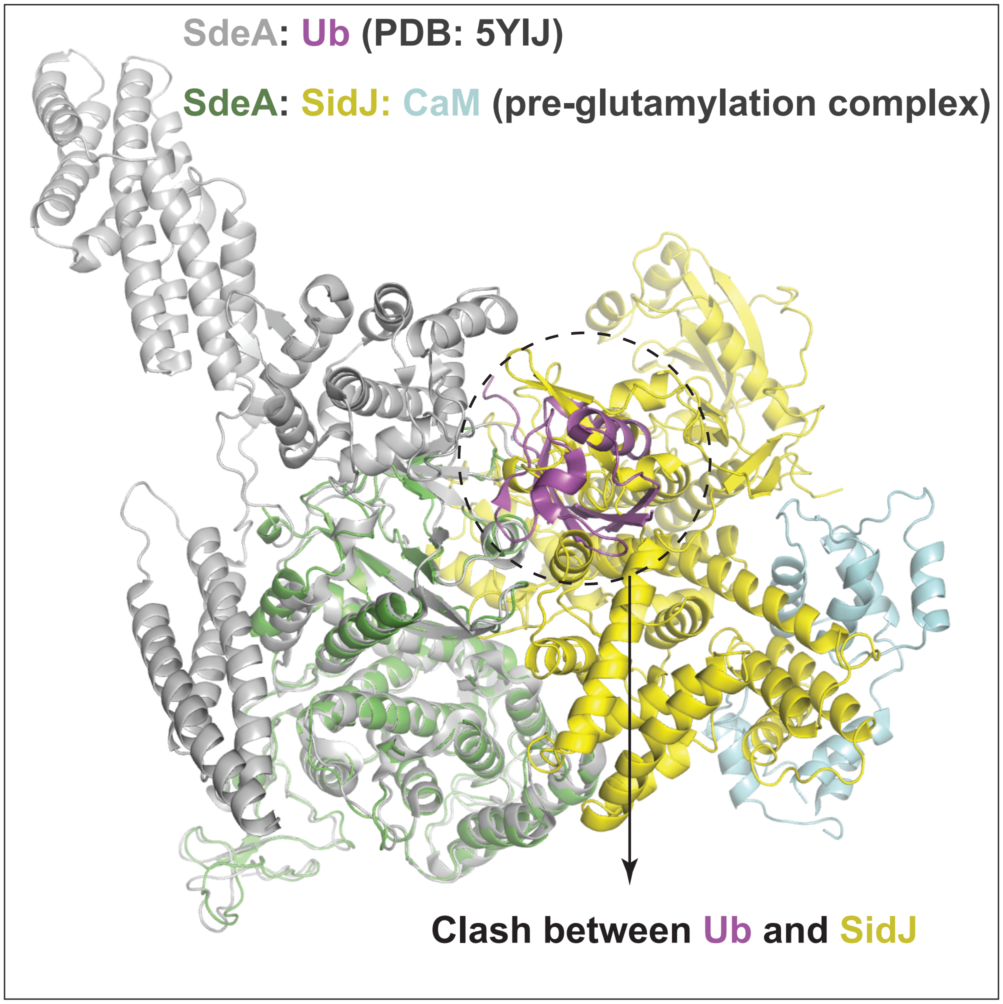

Supplementary Figure 13 – A tentative model for the catalysis of SidJ

Based on the previous studies and our mass spectrometry, biochemistry data shown in Figure 5 and Supplementary Figures 6-9, a tentative model for the catalysis of SidJ is proposed. According to the model, SidJ hydrolyzes ATP in the canonical pocket and gets autoAMPylated on the residue Lys370. Based on the importance of Lys370 in SidJ-mediated SdeA adenylylation and glutamylation (Figure 5E-F), we propose that autoAMPylation of Lys370 serves as an intermediate for SdeA adenylylation. But the exact mode of binding between SidJ and SdeA during SdeA adenylylation is unknown. Lys370 is essential for the AMPylation of Glutamates in the bridging peptide (Figure 5D), therefore we propose that AMPylation of Lys370 serves as a precursor to SidJ autoAMPylation on the bridging peptide. Bridging peptide residues are not important for SidJ-mediated adenylylation and glutamylation of SdeA (Figure 5E-F) therefore the modification of bridging peptide residues with AMP could be a side reaction of SidJ. Adenylylated SdeA binds to the migrated pocket of SidJ. An incoming L-Glutamate carries out SidJ-enabled nucleophilic attack on the acyl-phosphate link of adenylylated E860 of SdeA which results in the glutamylation of SdeA. The mode of L-Glutamate binding to SidJ's migrated pocket is modeled by Osinski et al., 2021 which shows that R522 plays a key role in L-glutamate binding along with R500 in the migrated pocket. After the glutamylation of SdeA, AMP group leaves and SidJ's migrated pocket becomes flexible and ready for the next cycle of reaction.

Supplementary Figure 13

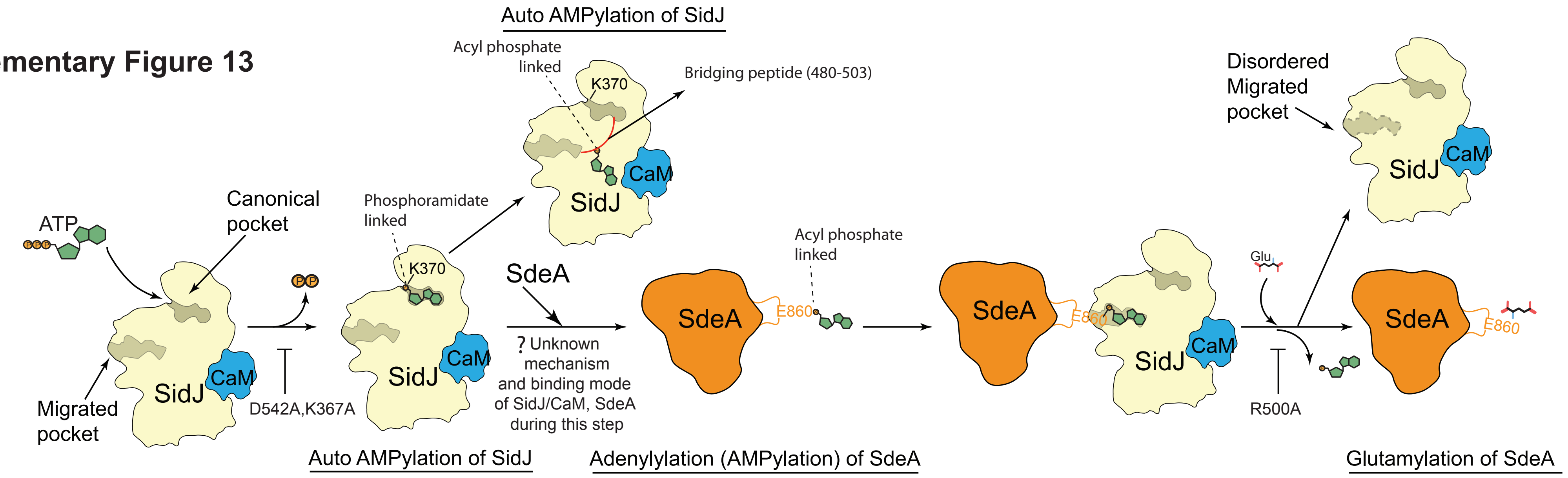

Supplementary Table 1 – cryo-EM data collection and processing parameters.

	**Pre-Glutamylation**	**Post-Catalytic**
Magnification	165k	120k
Voltage (kV)	300	200
Electron exposure (e-/Å^2)	47.45	35.0
Defocus range (μm)	-0.70 to -1.70 (0.10 steps)	-0.75 to -2.5 (0.25 steps)
Pixel size (Å)	0.504	0.941
Symmetry imposed	C1	C1
Initial particle images (no.)	2.683.400	635.561
Final particle images (no.)	140.022	58.448
Map resolution (Å)	2.94	3.71
FSC threshold	0.143	0.143
Map resolution range (Å)	2.9-6.0	3.7-8.0
**Refinement**		
	**Pre-Glutamylation**	**Post-Catalytic**
Refinement resolution limit (Å)	2.9	3.7
Model resolution range (Å)	2.9-6.0	3.7-8.0
Map sharpening B factor (Å^2)	-54.27	-72.3
Initial models used (PDB)	6OQQ; 5YIM	Pre-Glutamylation complex
**Model composition**		
Non-hydrogen atoms	10990	10746
Protein residues	1382	1365
Ligands	3	2
**B factors (Å^2)**		
Protein	63.85	53.32
Ligand	45.84	42.83
**R.m.s. deviations**		
Bond lengths (Å)	0.006 (0)	0.006 (0)
Bond angles (degrees)	0.607 (1)	0.676 (0)
**Validation**		
	**Pre-Glutamylation**	**Post-Catalytic**
MolProbity score	2.20	1.98
Clashscore	7.57	12.09
Rotamer Outliers (%)	3.41	0.09
**Ramachandran plot (%)**		
Favored	94.43	94.36
Allowed	5.57	5.64
Disallowed	0.00	0.00
**Deposition**		
	**Pre-Glutamylation**	**Post-Catalytic**
EMDB	EMD-13583	EMD-13591
PDB	7PPO	7PQE

Supplementary Table 2 – Primers used for site-directed mutagenesis

Mutation site	Forward primer	Reverse primer
SidJ_E565A	gatatcctcctgtaagcagtgcggagaagtaatgttttgtg	cacaaaacattacttctccgcactgcttacaggaggatatc
SidJ_H492A	cgtcttcaccaaaatgagtagcgaagatatcggccaattggg	cccaattggccgatatcttcgctactcattttggtgaagacg
SidJ_K367A	ggctctcctctttttttgtactgcgactgcaaccacatcattttg	caaaatgatgtggttgcagtcgcagtacaaaaaaagaggagagcc
SidJ_D542A	agactgtctcccaaagcagccagaccactgc	gcagtggtctggctgctttgggagacagtct
SidJ_K370A	cttaggctctcctcttgcttgtactttgactgcaaccacatcattt	aaatgatgtggttgcagtcaaagtacaagcaagaggagagcctaag
SidJ_Q350A	ctaaacagtaaagttctgcccgcgatacgcaataaacgatcact	agtgatcgtttattgcgtatcgcgggcagaactttactgtttag
SidJ_T353A	gtgtactaaacagtaaagctctgccctggatacgca	tgcgtatccagggcagagctttactgtttagtacac
SidJ_Y452A	ctgatttttatcgtgtagggcagtgaaataagttagaggggctttataaacatac	gtatgtttataaagcccctctaacttatttcactgccctacacgataaaaatcag
SidJ_N733A	tgggctttccctggcatatccacccaagtcctgatgag	ctcatcaggacttgggtggatatgccagggaaagccca
SidJ_N733L	ggcactcatcaggacttgggtggatatctaagggaaagcccatt	aatgggctttcccttagatatccacccaagtcctgatgagtgcc
SidJ_E497A	tgtcttcgcgctcgtctgcaccaaaatgagtatgg	ccatactcattttggtgcagacgagcgcgaagaca
SidJ_E499A	ttcctttgtcttcgcgcgcgtcttcaccaaaatga	tcattttggtgaagacgcgcgcgaagacaaaggaa
SidJ_E497A _E499A_E501A	ggtatcttcctttgtctgcgcgcgcgtctgcaccaaaatgagtatgg	ccatactcattttggtgcagacgcgcgcgcagacaaaggaagatacc
SidJ_E501A	gtatcttcctttgtctgcgcgctcgtcttcacc	ggtgaagacgagcgcgcagacaaaggaagatac
SidJ_R293A	ggtggataatctttcagcaaacataggattaaagctggagtcct	aggactccagctttaatcctatgtttgctgaaagattatccacc
SidJ_M696A	ataaatgctgtattgctccgcttgcagtgtatcttcatccaacttgg	ccaagttggatgaagatacactgcaagcggagcaatacagcatttat
SidJ_Y699A	ctcaccggaataaatgctggcttgctccatttgcagtgtatc	gatacactgcaaatggagcaagccagcatttattccggtgag
SidJ_△291-300	ggactccagctttaatcctGGCtatcattccactctgaccg	cggtcagagtggaatgataGCCaggattaaagctggagtcc
SidJ_Q259A	gacagaagagtccctgaattttgctacaaaggcgctttggtttttg	caaaaaccaaagcgcctttgtagcaaaattcagggactcttctgtc
SidJ_R262A	gaaagacagaagagtccgcgaatttttgtacaaaggcgctttgg	ccaaagcgcctttgtacaaaaattcgcggactcttctgtctttc
SidJ_F518A	ccatttatcaatgcgccctaattgggcctgtaaaacattcaaaagttgaacc	ggttcaacttttgaatgttttacaggcccaattagggcgcattgataaatgg
SidJ_L516A	cgccctaattggaactgtgcaacattcaaaagttgaaccaatgcctg	caggcattggttcaacttttgaatgttgcacagttccaattagggcg
SidJ_R500A	gtatcttcctttgtcttcggcctcgtcttcaccaaaatga	tcattttggtgaagacgaggccgaagacaaaggaagatac
SidJ_T493A	cgtcttcaccaaaatgagcatggaagatatcggccaat	attggccgatatcttccatgctcattttggtgaagacg
SidJ_Y732A	taatgggctttccctgttagctccacccaagtcctgatga	tcatcaggacttgggtggagctaacagggaaagcccatta
SdeA_T236D	taacggtatcatcatcatataatgaaaaccccatgggcccctg	caggggcccatggggttttcattatatgatgatgataccgtta
SdeA_T236L	gctttaacggtatcatctagatataatgaaaaccccatgggcccctgg	ccaggggcccatggggttttcattatatctagatgataccgttaaagc
SdeA_T236A	aacggtatcatcagcatataatgaaaaccccatgggcc	ggcccatggggttttcattatatgctgatgataccgtt
SdeA_F566A	ctgtttgtaattgtttcaaacaagcatcaacatcgttttcgcatcgtgctct	agagcacgatgcgaaaacgatgttgatgcttgtttgaaacaattacaaacag
SdeA_D833A	tttcaaagatgacattggaggcccaggtttcttttacaagc	gcttgtaaaagaaacctgggcctccaatgtcatctttgaaa
SdeA_K883A	gattttcccctttttgcgttgcaccatcaagggtgaccttga	tcaaggtcacccttgatggtgcaacgcaaaaaggggaaaatc
SdeA_E830A	tggagtcccaggttgcttttacaagcttaatctccg	cggagattaagcttgtaaaagcaacctgggactcca
SdeA_L827A	agtcccaggtttcttttacagccttaatctccgtggttgtac	gtacaaccacggagattaaggctgtaaaagaaacctgggact
SdeA_K826A	ggtttcttttacaagcgcaatctccgtggttgtactggcattg	caatgccagtacaaccacggagattgcgcttgtaaaagaaacc
SdeA_Q851A	cgccatggcgtcctactgccttggaatgcagcaaac	gtttgctgcattccaaggcagtaggacgccatggcg
SdeA_Q851L	ccatggcgtcctactagcttggaatgcagca	tgctgcattccaagctagtaggacgccatgg
