# Supplementary material for: Structural basis for protein glutamylation by the Legionella pseudokinase SidJ
Source: Nat Commun. 2021 Oct 26;12:6174. doi: 10.1038/s41467-021-26429-y (PMC8548325; doi:10.1038/s41467-021-26429-y)
Supplement: Supplementary file 3 — Reporting summary [file 41467_2021_26429_MOESM3_ESM.pdf]

nature research

Corresponding author(s): Sagar Bhogaraju

Last updated by author(s): 16-09-2021

Reporting Summary

Nature Research wishes to improve the reproducibility of the work that we publish. This form provides structure for consistency and transparency in reporting. For further information on Nature Research policies, see our Editorial Policies and the Editorial Policy Checklist.

Statistics

For all statistical analyses, confirm that the following items are present in the figure legend, table legend, main text, or Methods section.

n/a	Confirmed	
☐	☒	The exact sample size (n) for each experimental group/condition, given as a discrete number and unit of measurement
☐	☒	A statement on whether measurements were taken from distinct samples or whether the same sample was measured repeatedly
☒	☐	The statistical test(s) used AND whether they are one- or two-sided<br>*Only common tests should be described solely by name; describe more complex techniques in the Methods section.*
☒	☐	A description of all covariates tested
☒	☐	A description of any assumptions or corrections, such as tests of normality and adjustment for multiple comparisons
☐	☒	A full description of the statistical parameters including central tendency (e.g. means) or other basic estimates (e.g. regression coefficient) AND variation (e.g. standard deviation) or associated estimates of uncertainty (e.g. confidence intervals)
☒	☐	For null hypothesis testing, the test statistic (e.g. F, t, r) with confidence intervals, effect sizes, degrees of freedom and P value noted<br>*Give P values as exact values whenever suitable.*
☒	☐	For Bayesian analysis, information on the choice of priors and Markov chain Monte Carlo settings
☒	☐	For hierarchical and complex designs, identification of the appropriate level for tests and full reporting of outcomes
☒	☐	Estimates of effect sizes (e.g. Cohen's d, Pearson's r), indicating how they were calculated

Our web collection on statistics for biologists contains articles on many of the points above.

Software and code

Policy information about availability of computer code

Data collection	ImageLab 5.2.1, EPU cryo-EM software
Data analysis	ImageLab 5.2.1, GraphPad Prism 8, Relion 3.1, WARP 1.0.9, MaxQuant 1.6.5, Adobe Illustrator CC 2021, UCSF Chimera 1.15, PyMol2, CCP-EM 1.5.0, Phenix 1.14, Coot 0.9.5,

For manuscripts utilizing custom algorithms or software that are central to the research but not yet described in published literature, software must be made available to editors and reviewers. We strongly encourage code deposition in a community repository (e.g. GitHub). See the Nature Research guidelines for submitting code & software for further information.

Data

Policy information about availability of data

All manuscripts must include a data availability statement. This statement should provide the following information, where applicable:

- Accession codes, unique identifiers, or web links for publicly available datasets
- A list of figures that have associated raw data
- A description of any restrictions on data availability

All measurements taken for Acyl adenylate formation assays, Glutamylation kinetics assays, and Pyrophosphate release assays are available in the provided Source_data.xlsx file. All chromatograms shown are also available in the same file. Mass spectrometry data was submitted to the PRIDE database under accession code PXD028638. Cryo-EM maps for catalytic intermediate and post-catalytic structures were submitted to EMDB under accession codes EMD-13583 and EMD-13591, and the corresponding models were submitted to the PDB under accession codes 7PPO and 7PQE, respectively.

Field-specific reporting

Please select the one below that is the best fit for your research. If you are not sure, read the appropriate sections before making your selection.

- [x] Life sciences
- [ ] Behavioural & social sciences
- [ ] Ecological, evolutionary & environmental sciences

For a reference copy of the document with all sections, see nature.com/documents/nr-reporting-summary-flat.pdf

Life sciences study design

All studies must disclose on these points even when the disclosure is negative.

Sample size	No sample size calculation was done. All biochemical assays were performed in triplicates. To ensure the validity of the results shown, all assays were further repeated independently.
Data exclusions	No data was excluded from analysis
Replication	All data shown have been repeated at least twice with consistent, similar results. We have included detailed protocols in the methods section in order to ensure reproducibility of the results.
Randomization	All assays were performed using identical conditions in order to test for the effect of individual point mutations. Therefore, no randomization was necessary in our experiments, and was thus not performed.
Blinding	Because this study aims to characterize the catalytic cycle of SidJ with no clinical implications tested, blinding was not necessary in our experiments, and was thus not performed.

Reporting for specific materials, systems and methods

We require information from authors about some types of materials, experimental systems and methods used in many studies. Here, indicate whether each material, system or method listed is relevant to your study. If you are not sure if a list item applies to your research, read the appropriate section before selecting a response.

Materials & experimental systems

n/a	Involved in the study	
[ ]	[x]	Antibodies
[ ]	[x]	Eukaryotic cell lines
[x]	[ ]	Palaeontology and archaeology
[x]	[ ]	Animals and other organisms
[x]	[ ]	Human research participants
[x]	[ ]	Clinical data
[x]	[ ]	Dual use research of concern

Methods

n/a	Involved in the study	
[x]	[ ]	ChIP-seq
[x]	[ ]	Flow cytometry
[x]	[ ]	MRI-based neuroimaging

Antibodies

Antibodies used	GFP (B-2) (Cat # sc-9996, Lot# I2418, provider: Santa Cruz Biotechnology, Dilution: 1:2000)<br>DSRed2 (Cat # sc-101526, Lot# L1714, provider: Santa Cruz Biotechnology, Dilution: 1:1000)
Validation	GFP (B-2) Santa Cruz Biotechnology (sc-9996)<br>Validation statement from the manufacturer: recommended for detection of GFP and GFP mutant fusion proteins by WB, IP, IF, FCM and ELISA.<br>Validation found at provider's website- https://www.scbt.com/p/gfp-antibody-b-2<br>DSRed2 Santa Cruz Biotechnology (sc-101526)<br>Validation statement from the manufacturer: recommended for detection of DsRed2 by WB and ELISA.<br>Validation found at provider's website- https://www.scbt.com/p/dsred2-antibody-25

Eukaryotic cell lines

Policy information about cell lines

Cell line source(s)	HEK293T (ATCC® CRL-3216™)
Authentication	Cell lines were authenticated using STR DNA profiling.
Mycoplasma contamination	All the cell lines used tested negative for mycoplasma.

Commonly misidentified lines<br>(See ICLAC register)	The cell lines used in the study are not in the commonly misidentified lines list.
