# Supplementary material for: Structural basis for protein glutamylation by the Legionella pseudokinase SidJ
Source: Nat Commun. 2021 Oct 26;12:6174. doi: 10.1038/s41467-021-26429-y (PMC8548325; doi:10.1038/s41467-021-26429-y)

Fig. 1D - Autoradiography

Triplicate experiment used for quantification

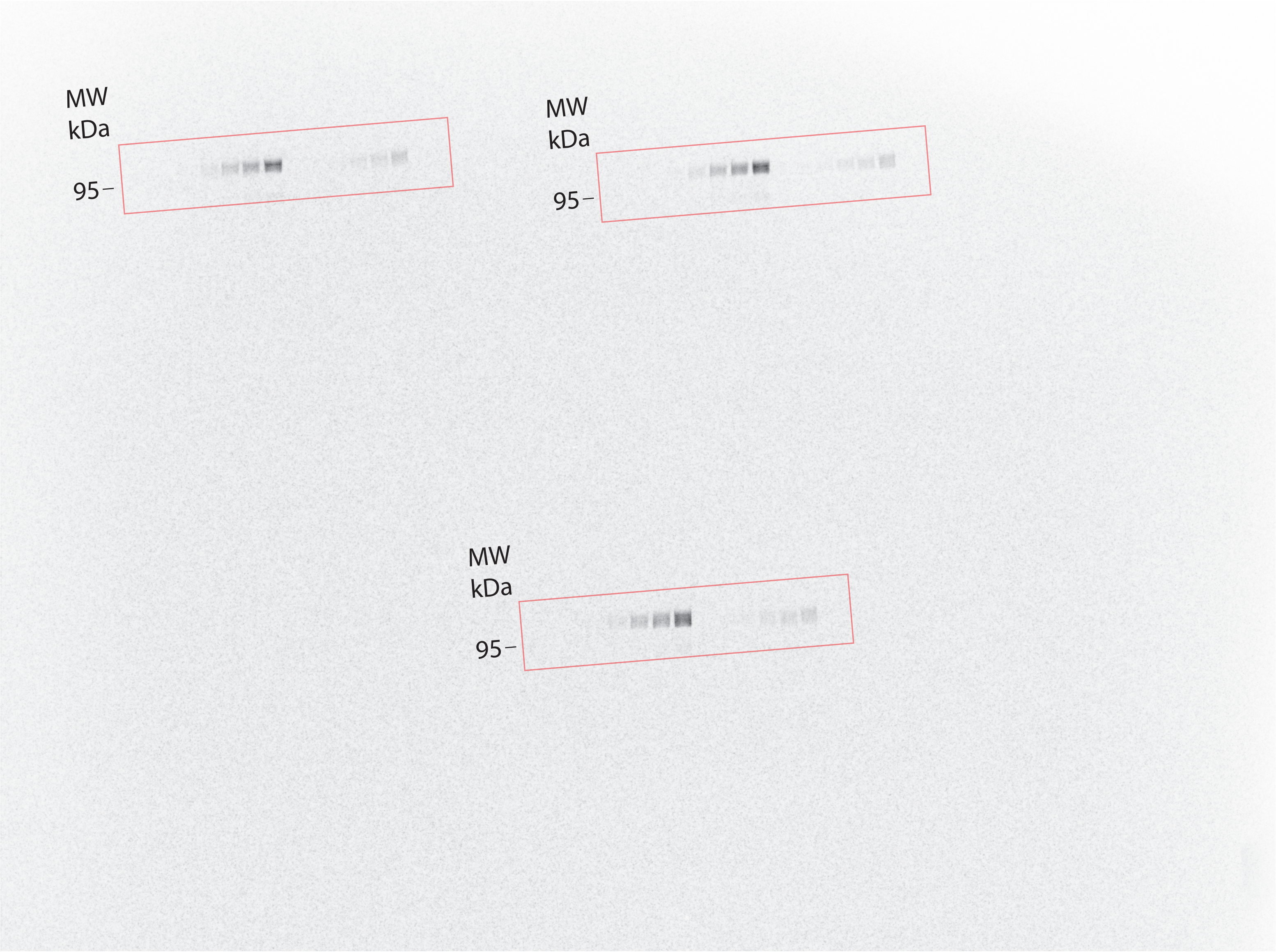

Fig. 2D - Autoradiography

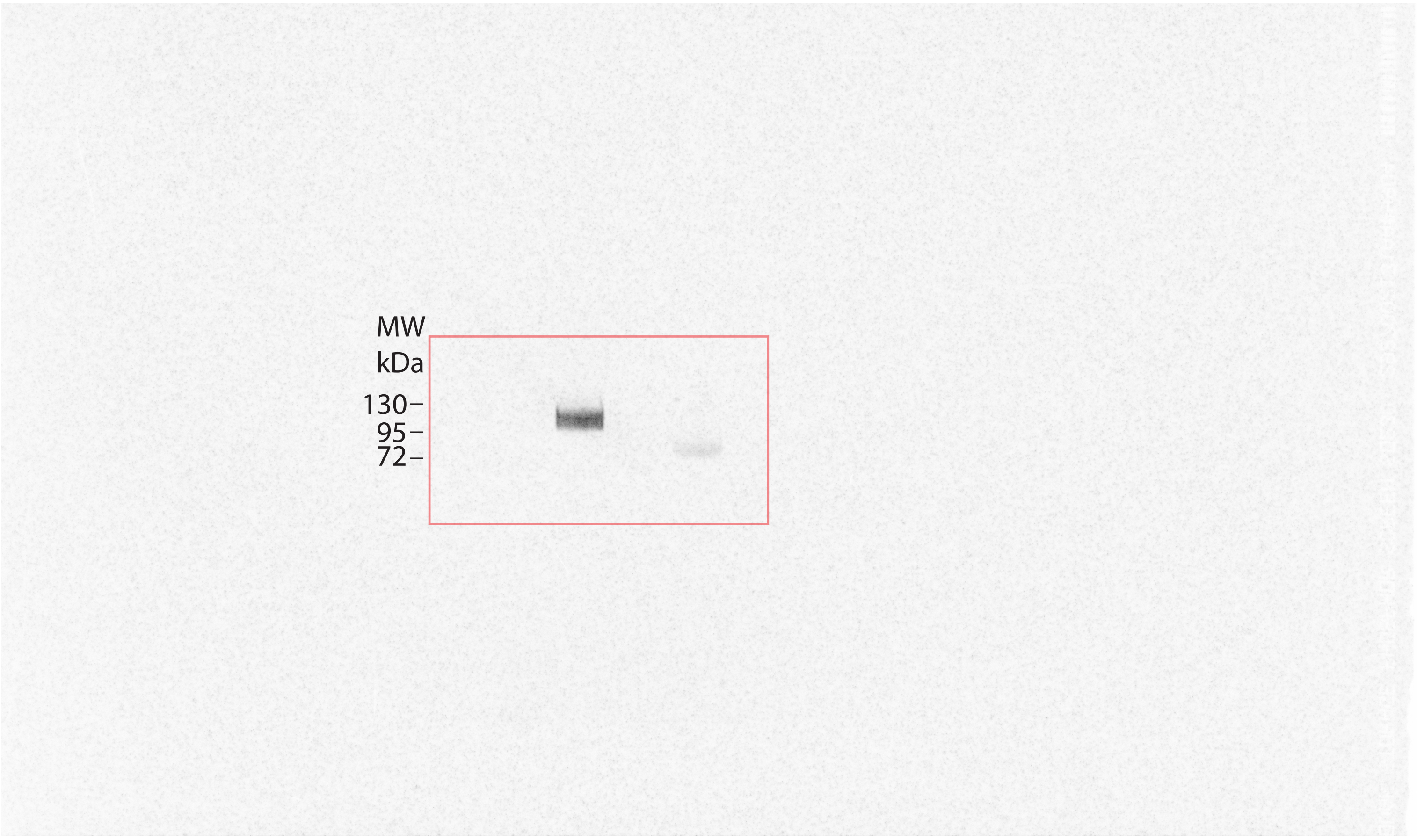

Fig. 3D - Autoradiography

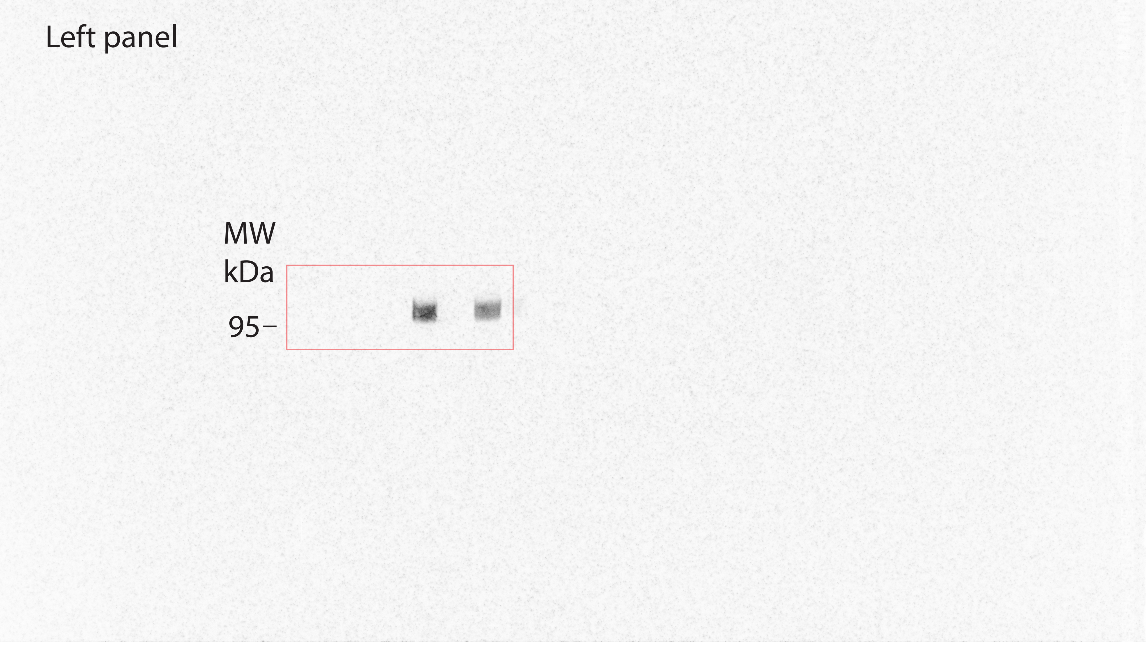


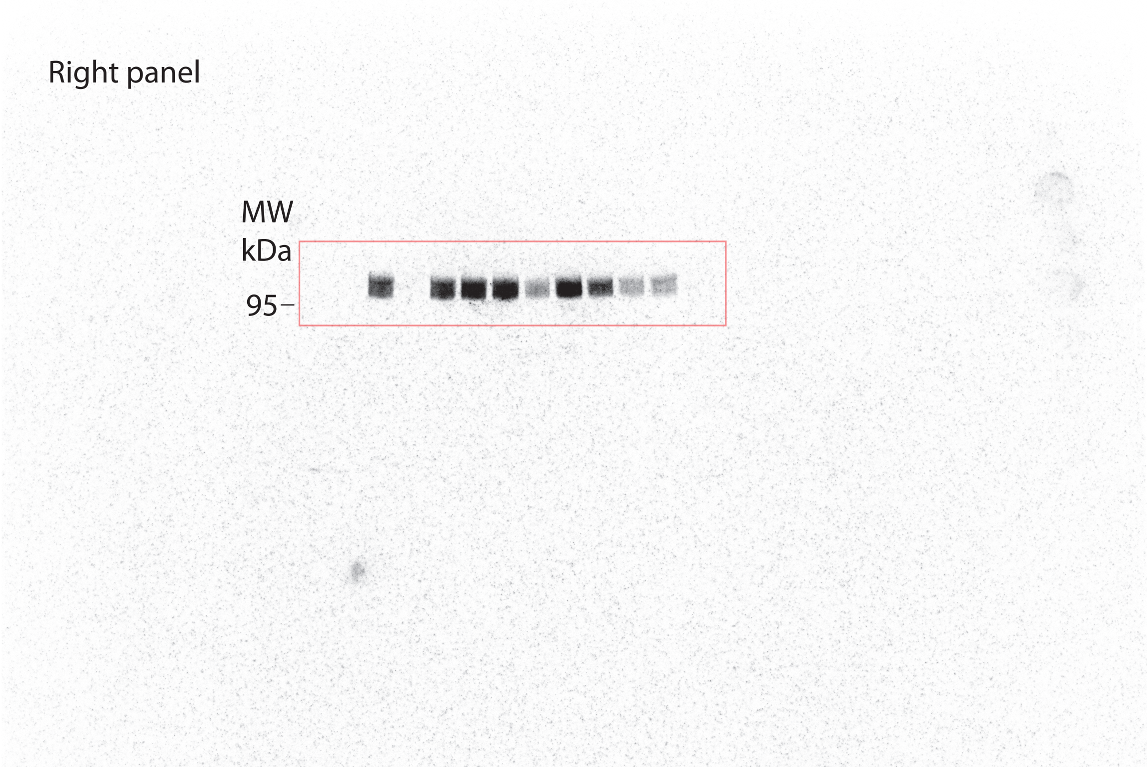

Fig. 3F - Autoradiography

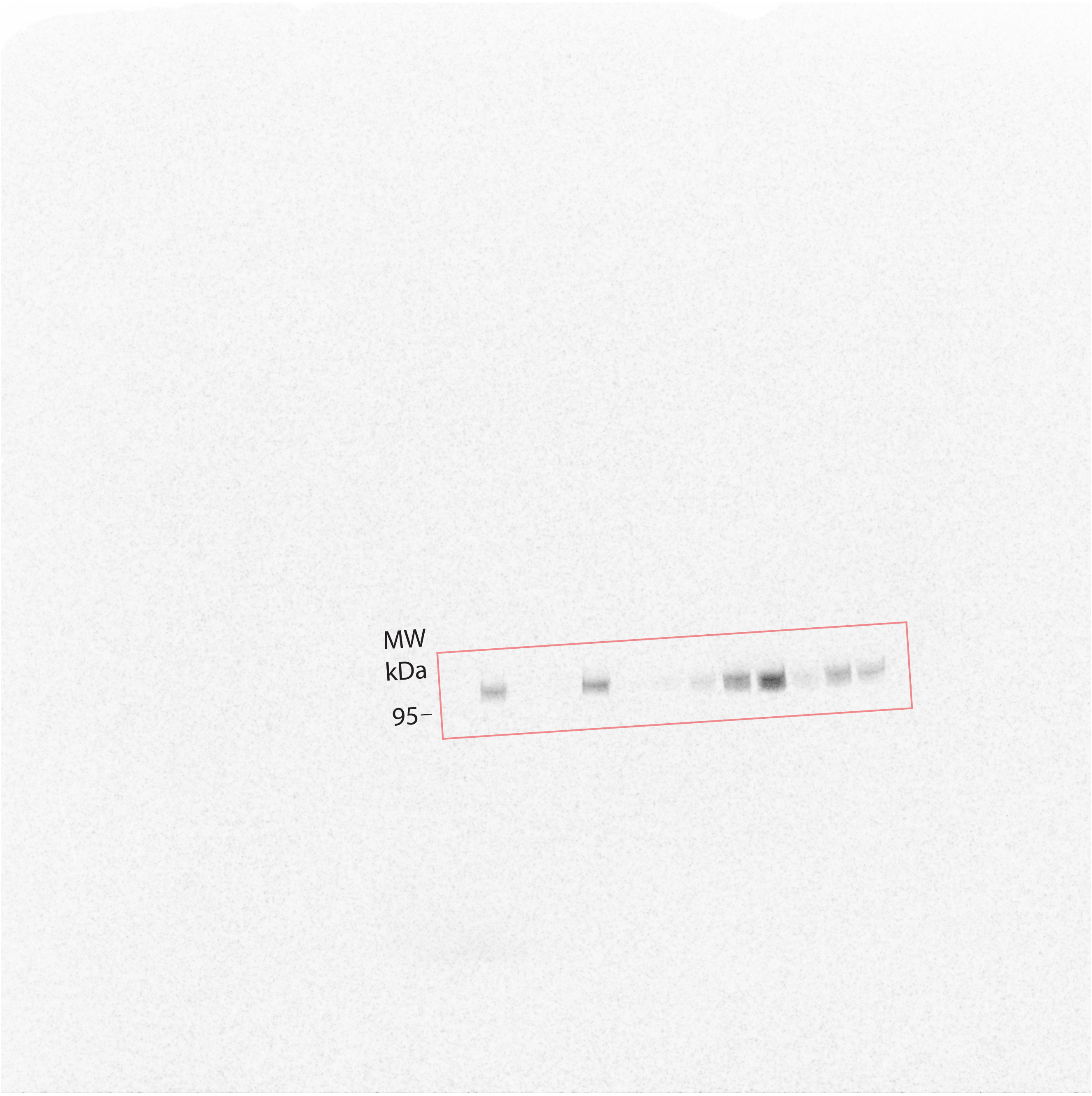

Fig. 4B - Autoradiography

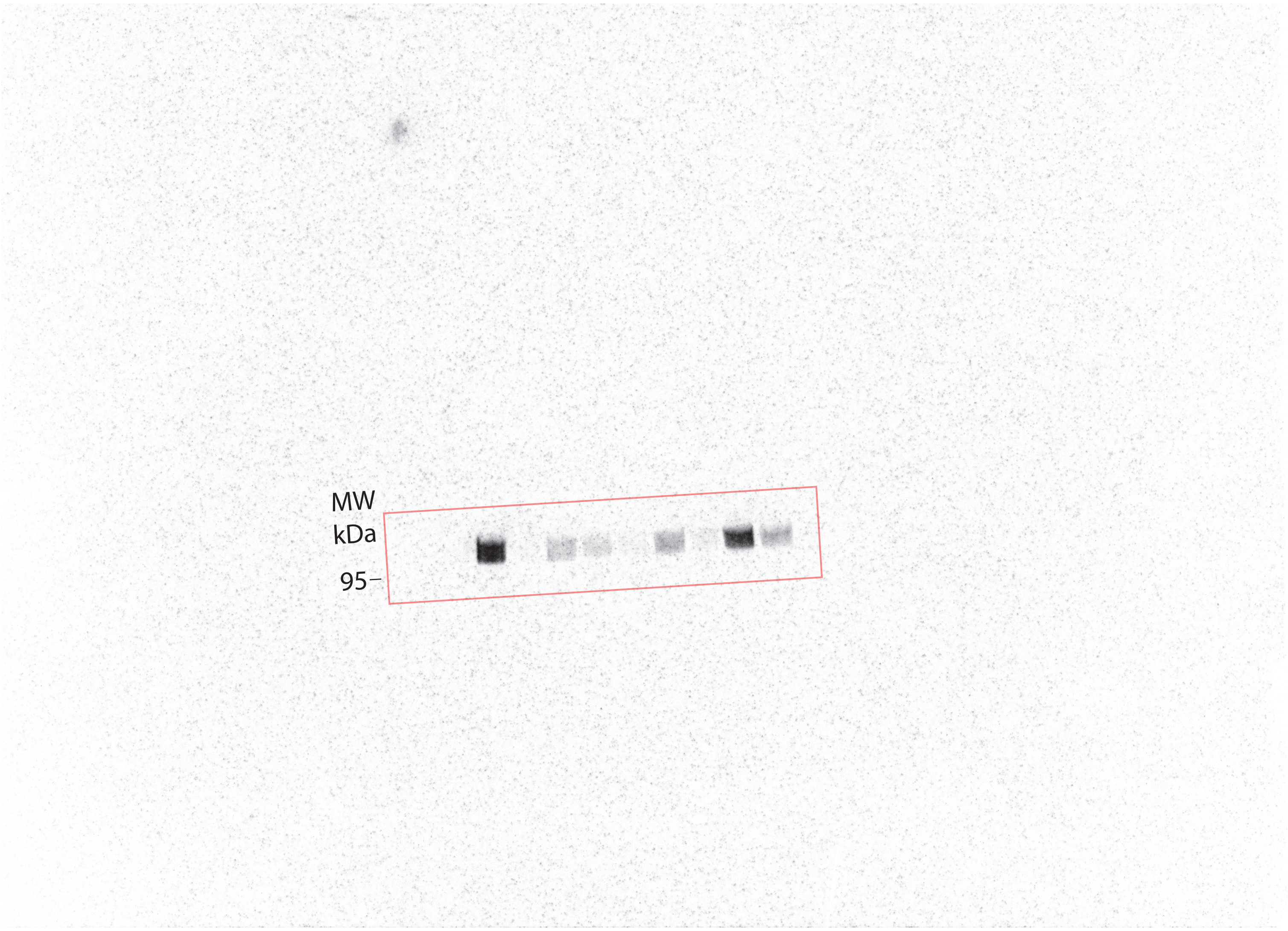

Fig. 5A, Supp. Fig. 6B - Autoradiography

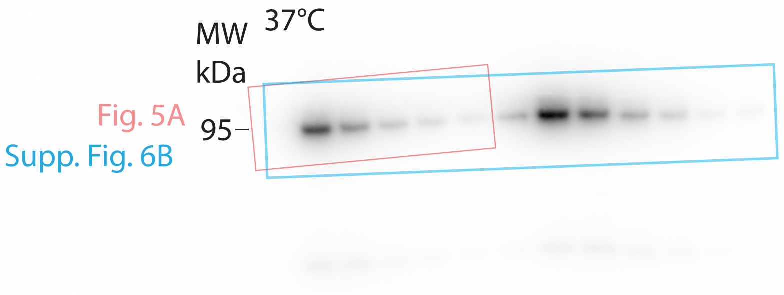


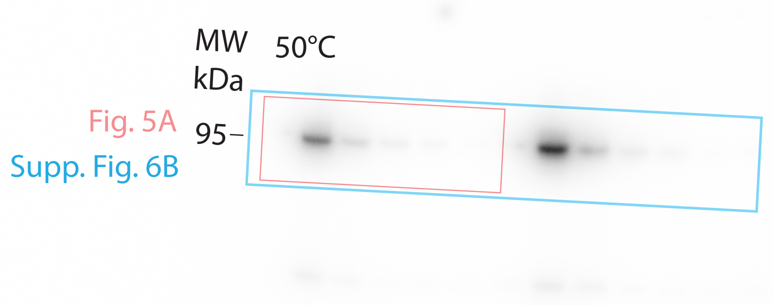

Fig. 5D, 5F - Autoradiography

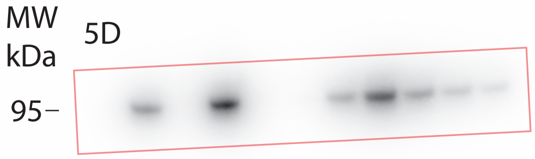


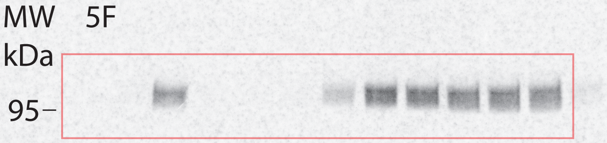

Supp. Fig. 1A - Western Blot

Anti-GFP

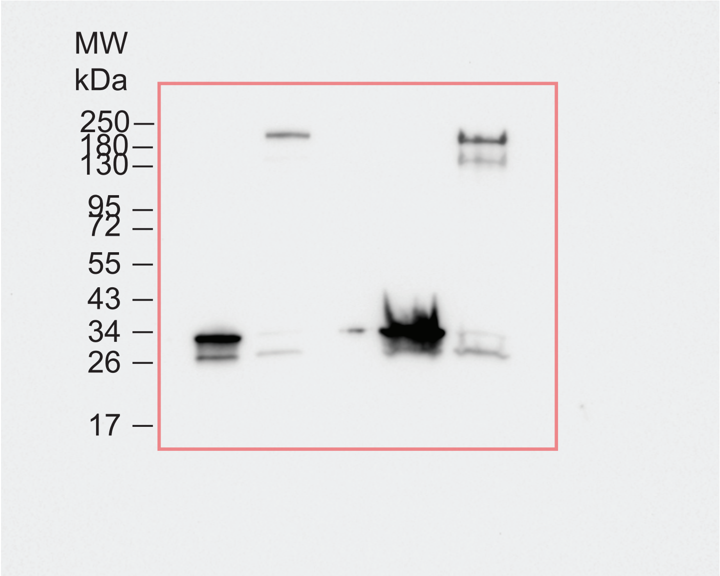


Anti-mCherry

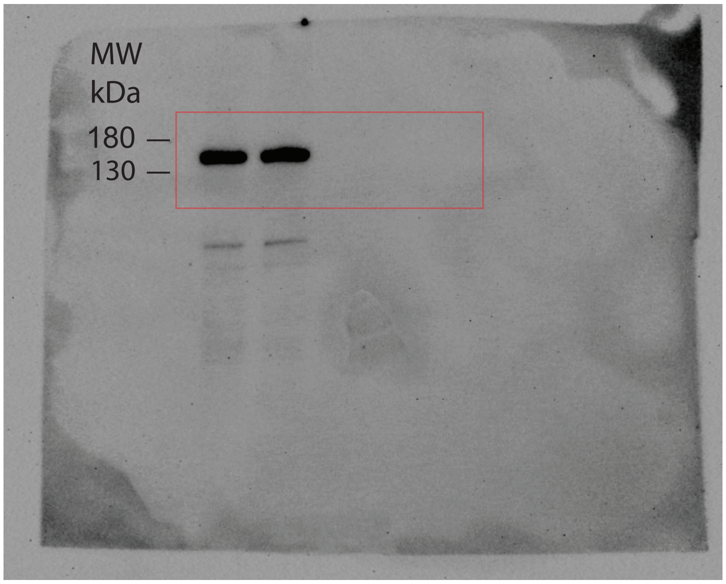

Supplement: Supplementary file 4 — Source Data [file 41467_2021_26429_MOESM4_ESM.zip › Source_data/Uncropped_gels.pdf]
