# Supplementary material for: Structural basis for protein glutamylation by the Legionella pseudokinase SidJ
Source: Nat Commun. 2021 Oct 26;12:6174. doi: 10.1038/s41467-021-26429-y (PMC8548325; doi:10.1038/s41467-021-26429-y)
Supplement: Supplementary file 2 — Peer Review File [file 41467_2021_26429_MOESM2_ESM.pdf]

nature portfolio

Peer Review File

Structural basis for protein glutamylation by the Legionella pseudokinase SidJ

Open Access This file is licensed under a Creative Commons Attribution 4.0 International License, which permits use, sharing, adaptation, distribution and reproduction in any medium or format, as long as you give appropriate credit to the original author(s) and the source, provide a link to the Creative Commons license, and indicate if changes were made. In the cases where the authors are anonymous, such as is the case for the reports of anonymous peer reviewers, author attribution should be to 'Anonymous Referee' followed by a clear attribution to the source work. The images or other third party material in this file are included in the article's Creative Commons license, unless indicated otherwise in a credit line to the material. If material is not included in the article's Creative Commons license and your intended use is not permitted by statutory regulation or exceeds the permitted use, you will need to obtain permission directly from the copyright holder. To view a copy of this license, visit http://creativecommons.org/licenses/by/4.0/.

Editorial Note: This manuscript has been previously reviewed at another journal that is not operating a transparent peer review scheme. This document only contains reviewer comments and rebuttal letters for versions considered at *Nature Communications*.

REVIEWERS' COMMENTS

Reviewer #1 (Remarks to the Author):

The authors have adequately addressed the questions I raised in previous review. I am satisfied with this new version. It is a beautiful piece of work, which reveals the molecular mechanism of an unusual type of protein glutamylation.

Reviewer #2 (Remarks to the Author):

The authors have significantly revised the manuscript, which is markedly improved. The addition of new data that clarifies certain mechanistic steps is an improvement. The revisions to the text are also helpful in terms of clarity and rigor. I feel that it is acceptable for publication.

Reviewer #3 (Remarks to the Author):

In the revised version the authors address adequately my comments and added additional information and figures to the manuscript. I have no further comments and recommend acceptance of the manuscript for publication.
